# Study of the Total Antioxidant Capacity (TAC) in Native Cereal−Pulse Flours and the Influence of the Baking Process on TAC Using a Combined Bayesian and Support Vector Machine Modeling Approach

**DOI:** 10.3390/foods12173208

**Published:** 2023-08-25

**Authors:** Daniel Rico, Ana Belén Cano, Sergio Álvarez Álvarez, Gustavo Río Briones, Ana Belén Martín Diana

**Affiliations:** Agrarian Technological Institute of Castilla and Leon (ITACyL), Ctra. Burgos Km 119, Finca Zamadueñas, 47071 Valladolid, Spain; ricbarda@itacyl.es (D.R.); alvalvse@itacyl.es (S.Á.Á.); ita-riobrigu@itacyl.es (G.R.B.)

**Keywords:** pulses, antioxidant capacity, prediction, thermal processing, flour, Bayesian model, cereals, support vector machines (SVM)

## Abstract

During the last few years, the increasing evidence of dietary antioxidant compounds and reducing chronic diseases and the relationship between diet and health has promoted an important innovation within the baked product sector, aiming at healthier formulations. This study aims to develop a tool based on mathematical models to predict baked goods’ total antioxidant capacity (TAC). The high variability of antioxidant properties of flours based on the aspects related to the type of grain, varieties, proximal composition, and processing, among others, makes it very difficult to innovate on food product development without specific analysis. Total phenol content (TP), oxygen radical absorbance capacity (ORAC), and ferric-reducing antioxidant power assay (FRAP) were used as markers to determine antioxidant capacity. Three Bayesian-type models are proposed based on a double exponential parameterized curve that reflects the initial decrease and subsequent increase as a consequence of the observed processes of degradation and generation, respectively, of the antioxidant compounds. Once the values of the main parameters of each curve were determined, support vector machines (SVM) with an exponential kernel allowed us to predict the values of TAC, based on baking conditions (temperature and time), proteins, and fibers of each native grain.

## 1. Introduction

Cereals and legumes are common staple foods. These products represent an important source of nutrients from the diet and have an irreplaceable role in nutrition [1]. Cereals are frequently consumed as baked goods, such as bread or biscuits, among other products, with legumes gaining importance as total or partial substitutes for cereal flour in those types of products. In the 1990s, different aspects such as industrialization and changes in lifestyles, among other factors, favored the production of under-nourishing baked goods based on refined flours, rich in sugars, and saturated fatty acids, with poor fiber and naturally present antioxidants.

Traditionally, fruits and vegetables have been identified as major sources of dietary antioxidants, but grains have gained interest due to the proven inverse association between whole grain consumption and the risk of chronic diseases, such as cardiovascular diseases, diabetes, and cancer [2,3,4,5], due to their high content in fiber and free and bound phenolic compounds [6,7].

During the last few years, the increasing awareness by consumers of the relationship between diet and health and the industrial innovation to satisfy consumer demands have driven the baked-product sector to work on more nutritional and healthier formulations. The market for natural antioxidants, with high demand not only in the food sector but with other applications such as cosmetics and plastic, is expected to grow from USD 321.4 million in 2022 to USD 409.7 million in 2032, at a CAGR of 2.4% [8].

Our body is naturally equipped with antioxidant mechanisms to control damage from ROS and RNS (reactive oxygen or nitrogen species); however, dietary antioxidants from food or nutritional supplements are believed to contribute to oxidative balance [9]. Moreover, these compounds affect food quality by avoiding lipid and protein oxidation.

Food bioactive molecules are characterized by their chemical diversity, which complicates the measurement of single-compound antioxidant capacity. The concept of total antioxidant capacity (TAC), including the synergic and redox interactions between the different molecules present in the food, was introduced to avoid these limitations. The appropriate use of TAC measurements provides support for the interpretation of complex phenomena and can be a tool for screening studies previous to in-depth research investigations [10].

The broad types of chemical structures of antioxidant compounds in food complex matrices make necessary the use of more than one measurement to assess their antioxidant capacity. One of the main aspects of selecting antioxidant assays is the reaction mechanism. There are two main mechanisms, single electron transfer (SET) and hydrogen atom transfer (HAT). In SET antioxidant reactions, the free radical loses its condition by “pairing” its unpaired electron. In HAT reactions, the free radical is electronically stabilized through a mechanism that involves the direct transfer of a hydrogen atom [11].

Different assays can be used to estimate the total capacity of foods to scavenge reactive oxygen and nitrogen species and free radicals or to assess the content of reducing compounds. Among the most popular assays, ABTS^+•^ (2,2′-azinobis-(3-ethylbenzothiazoline-6-sulfonic acid) and DPPH^•^ (2,2-diphenyl-1-picrylhydrazyl) radical reduction methods can be found; both can be reported with Trolox (6-hydroxy-2,5,7,8-tetramethylchroman-2-carboxylic acid) as standard. The ABTS^+•^ assay is effective in estimating hydrophilic and lipophilic antioxidants, while the DPPH^•^ assay has important limitations for lipophilic compounds. Although they are used widely in screening studies, both have limitations associated with oxygen, pH, or uncertain reaction kinetic endpoints, which can reduce their reproducibility. Also, both assays use synthetic radicals, which are not present in biological systems.

Oxygen radical absorbance capacity (ORAC) is another widely adopted method; in this case, although the radical initiator is a synthetic compound, the peroxyl radicals produced, against which antioxidant capacity is evaluated, are of relevance for food oxidation mechanisms [12]. Lipid and protein peroxidation have been linked to the pathogenesis of various diseases through different mechanisms, such as disturbance of membrane organization, functional loss of proteins and DNA, or accumulation of modified proteins in cells [13].

The reducing capacity is also important to assess the TAC; the ferric-reducing antioxidant power assay (FRAP) is based on the reduction of the 2,4,6-tripyridyl- -triazine (TPTZ)–Fe3+ to TPTZ-Fe2+ complex.

Wholegrain cereals and pulses contain a wide range of bioactive components with antioxidant effects, such as proteins, dietary fiber (TDF), and phenolic compounds present in free and bound forms [14,15,16], which contribute to product antioxidant capacity. The Folin−Ciocalteu assay is generally used to measure total phenol content (TPC); it is not a specific method for phenolic compounds, although it is a quick and effective method to evaluate them. It is important to highlight that this method can also respond to amines and reducing sugars, which can interfere with the real value; but also, it can be interesting in the case of processed foods, where Maillard reactions occur, as this method can evaluate the antioxidant capacity of Maillard reaction products (MRP) [17].

During the last few years (2010–2022), more than 8000 articles have been published on the antioxidant capacity in the food area [18]. However, there is a high variability of data published, which comes from different aspects such as the complexity of food matrices, raw material variability, lack of standards in the assays of the antioxidant capacity, and extraction methodologies, among others.

One strategy to overcome the difficulties of the variability in antioxidant capacity evaluation is relying on extensive databases [19], which can be used for comparison and reference. For example, USDA has developed two databases, one of reducing power values of more than 1100 food raw and processed products [20] and a second of ORAC values, with 326 different food items [21]. Another example would be the attempt to estimate the daily intake of hydrophilic antioxidants in the Japanese population by Takebayashi et al. [22].

The use of mathematical tools for assessing and predicting antioxidant properties of compounds can also be used as a strategy for food antioxidant estimation. QSAR models (quantitative structure-activity relationships) are used successfully to identify patterns in chemical structure data and correlate and predict the antioxidant capacity of compounds [23]. However, these studies have limited applications to food matrices, where complex mixtures of different compounds are found.

Support vector machine (SVM) methodology is extensively used in pattern recognition because of its capability to classify future unseen data and its good generalization performance [24]. SVM also has the ability to perform well with small datasets.

For this reason, the objective of this paper was to use the support vector machine (SVM) technique to develop a mathematical modeling tool based on proximal composition to predict total antioxidant capacity, as measured with three widely adopted methods integrating different aspects of antioxidant capacity assays (TP, ORAC, and FRAP). This work was intended to model the redox status of thermally-processed flours from different types of cereals and pulses and provide an estimation of TAC of baked cereal- and legume-based products to the food industry.

## 2. Materials and Methods

### 2.1. Raw Material

A total of 136 varieties of 10 different grain types (cereals and pulses) were provided by the experimental agronomic program (PEA) of ITACyL or obtained from local markets. The varieties provided by PEA were cultivated and harvested according to uniform protocols, which reduce variability associated with agronomic aspects. The varieties and grains were the following:

Wheat (Chambo, LGWF 16 1321, RW72009, LGWF17 118, LGWE 18 8199, LGWF 17 5114, LGWF 5114, CF 14255, RW 21837, RW 72010, RW 72003, RW 72003, Marcopolo, RW 21846, Filón, Camargo, FDN 18 WW 0240, Nogal, Berdun, LG WE 178300, Boticelli, HAW 18-001, RW21968, LGW 178302, RW 72006, RW71804, Candeal PANE 247, Candeal, and 12 commercial flours provided from local markets). Rye (Teodor, Igor, Loretto, Vinetto, Su Promotor, Petkus, Poseido, Arvid, Stannos, and two commercial flours provided from local markets. Corn (LG3490, PO937, LG31545, SY Caarioca, Urbanix, 47M, Berlioz, KWS Selecto, RGT Huxxo, and four commercial flours provided from local markets). Triticale (Kitsurf, Saleroso, Randam, Rivolt, RGTsuliac, Vivacio, RGT/Zaragozac., Trimour, Amarillo/105, LG Plutón, BELOTAC Cerraton (BU), RGT/KADJAC, RGT/COPLAC Cerraton, Bondadoso Cerraton). Oats (nine commercial flours provided from local markets). Rice (six commercial flours provided from local markets). Sorghum (White: KSH9635W, KSH8G26, KWS Octavius, KSH9G37W, KSH9G32W, P1288Y20, and Brown: KWS Nemesis, PR88P68, Shamal, Foehm, Es Alize). Chickpea (Tauriton, Sultano, Turi, Villaturiel, Urbel, Ituci, Krema, Maragia L., Reale L, Vulcano, Pirón, Kasin, and three commercial flours provided from local markets). Lentil (Guareña, Paula, and three commercial flours provided from local markets). Soybean (Sirocca+, Sirocca-, SB8, ES Pallador, Luna, Pepita, Es Isidor, Es Mentor, S/19/12, Primus, SB07, SB44, Proteix, Panoramix, Casleis).

### 2.2. Sample Preparation

Grain samples were reduced to uniform powder using a mill (Model Cyclotec 1093, Foss, Hilleroed, Denmark) fitted with a 0.5 mm screen, and then stored in sealed polyethylene/plastic bags in dark conditions to ensure stability until analysis.

**Figure 1:**
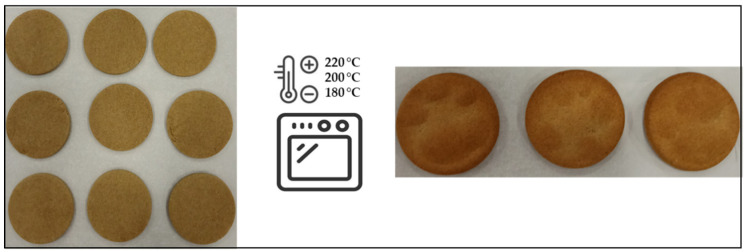
Biscuits prepared before and after thermal treatment (baking process) at different temperatures (180, 200, and 220 °C and times (0–1500 s).

Afterward, wheat and rye flours were used as models for the evaluation of thermal processing (temperature and time) on the antioxidant capacity (Figure 1). Biscuit was used as a model food. Biscuits were formulated with 10.17 g of flour, 3.85 g of sunflower oil, and 0.98 g of water. Each biscuit weighed 15 g. After kneading, the dough was cut using a round mold of Ø 9.5 cm and 0.5 cm in height. Biscuits were baked at temperatures of 180, 200, and 220 °C for 0–1500 s. The biscuits were frozen and lyophilized to stabilize the samples. After that, biscuits were milled to a particle size below 0.5 mm and stored in sealed polyethylene/plastic bags in dark conditions to ensure stability until analysis. The baking procedure for each sample, temperature and baking time, was carried out in duplicate with six biscuits. Each set of six biscuits was pooled for analysis.

### 2.3. Proximal Composition

Moisture content was measured gravimetrically by drying samples at 100 °C for 24 h. Total protein content was determined by the Dumas method (AOAC method 990.03) [25]. A conversion factor of 5.7 was used to calculate protein content from nitrogen values. Total fat content was determined using dried samples extracted with petroleum ether (BP 40–60 °C) for 4 h in a Soxtec fat extracting unit (AOAC 2005, method 2003.05) [25]. Ash content was determined by sample incineration in a muffle furnace at 550 °C for 5 h (AOAC 2005, method 923.03) [25]. Carbohydrates were estimated by difference. Total dietary fiber (TDF) content was evaluated using a kit provided by Sigma (TDF100A-1KT, St. Louis, MO, USA), in accordance with the manufacturer’s instructions, based on the AOAC method 985.29 [25]. All parameters were evaluated in duplicate. Proximal composition analysis was expressed in g 100 g^−1^ dry matter (d.m.).

### 2.4. Colorimetric Analysis

Color parameters lightness (L*), redness (a*), and yellowness (b*) were measured using a colorimeter (CM-2600d, Konica Minolta, Osaka, Japan) adjusted as D65 standard illuminate, 45/0 sensor, and 10° standard observer. The colorimeter was standardized using a light trap and a white calibration plate. Four measurements per sample were taken directly on the samples.

### 2.5. Extract Preparation

One gram of ground (mesh size 0.5 mm) sample was extracted with 8 mL of methanol: water (1:1, *v*/*v*; acidified to pH = 2 with 0.1 M HCl) in an orbital shaker (250 rpm, 30 min) using magnetic agitation. After centrifugation (2057× *g*, 10 min), the supernatant was collected and filtered (Filter lab paper n. 1249). The extraction was repeated three times. The combined methanol extracts were adjusted to 25 mL. All analyses were performed in duplicate. Extract aliquots were stored at −80 °C until further analysis.

### 2.6. Total Phenol (TP) Content

TP was measured using the Folin−Ciocalteu method as described by Slinkard and Singleton, with modifications [26]. The absorbance was measured at 765 nm with a microplate reader (Fluostar Omega, BMG, Ortenberg, Germany). Results were calculated using a calibration curve with gallic acid as standard (9.8–70 mM) and expressed as mg gallic acid equivalents (GAE) 100 g^−1^ sample (d.m.).

### 2.7. Oxygen Radical Absorbance Capacity (ORAC)

The procedure was based on a previously reported method by Ou et al. [27], with slight modifications. The standard curve of Trolox (7.5–180 mM) and samples were diluted in phosphate buffer (10 mM, pH 7.4). Fluorescence was monitored between 100 and 120 min with a microplate reader (Fluostar Omega, BMG, Ortenberg, Germany), using 485 nm excitation and 520 nm emission filters. Results were calculated using the areas under the fluorescein decay curves between the blank and the sample and expressed as µmol Trolox equivalents (TE) 100 g^−1^ sample (d.m.).

### 2.8. Ferric-Reducing Antioxidant Power (FRAP)

The procedure was based on a previously reported method by Benzie and Strain [28], with slight modifications. A 300 mM acetate buffer, pH 3.6 (mixing a solution of 300 mM sodium acetate and 300 mM glacial acetic acid until pH 3.6), a 10-mM TPTZ (2,4,6-tripyridyl-triazine) solution in 40 mM HCL, and a 20-mM FeCL_3_.6H_2_O solution were prepared. The FRAP working solution was prepared by mixing the acetate buffer, TPTZ solution, and FeCl_3_.6H_2_O solution in a volume ratio of 10:1:1. The absorbance was measured at 593 nm with a microplate reader (Fluostar Omega, BMG, Ortenberg, Germany) using a calibration curve with FeSO_4_.7H_2_O (100–1000 µM). The results were expressed as mmol Fe equivalents (FeE) 100 g^−1^ sample (d.m.).

### 2.9. Statistical Analysis and Mathematical Modelling

Chemical composition and antioxidant capacity were shown using average and standard deviation, ANOVA one-way analyses were carried out to find differences between groups, and results were displayed in stacked bars and boxplot graphs. In addition, prior centerd and standardized data, principal component analysis (PCA), and distributed stochastic neighbor embedding (t-SNE) were used to visualize the chemical composition profiles of the grains, and Spearman Rank correlation coefficients were performed to elucidate the relationships among chemical variables and antioxidant biomarkers. All statistical analyses were performed with both R (R: The R Project for Statistical Computing (r-project.org) and Python (Welcome to Python.org) software packages.

Modeling of the antioxidant capacity of the grains, using as output variables (TP, ORAC, and FRAP), was carried out considering as input variables the type of grain, proximal composition (protein and fiber), and baking temperature. Model development was performed with the RJAGS package (R Project). The maximization of R^2^ was used as criteria for model applicability evaluation, using models from the scikit-learn packages from Python and lme4, nlme, and e1071 libraries from R. Finally, a local sensitivity analysis (using the complete final model and varying input values) was carried out to assess the predictor importance and identify their effect on the curve response.

## 3. Results and Discussion

### 3.1. Compositional Characteristics of the Native Flours

**Figure 2:**
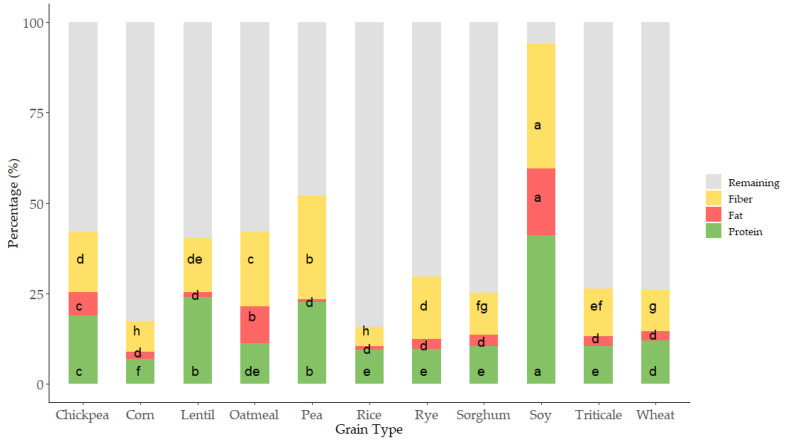
Stacked bar graph representing the proximal profile according to grain type. Letters of each color denote statistical differences between means (one-way ANOVA, posthoc Duncan’s test, *p* ≤ 0.05). Data was expressed in percentages (g 100 g^−1^ d.m.).

**Figure 3:**
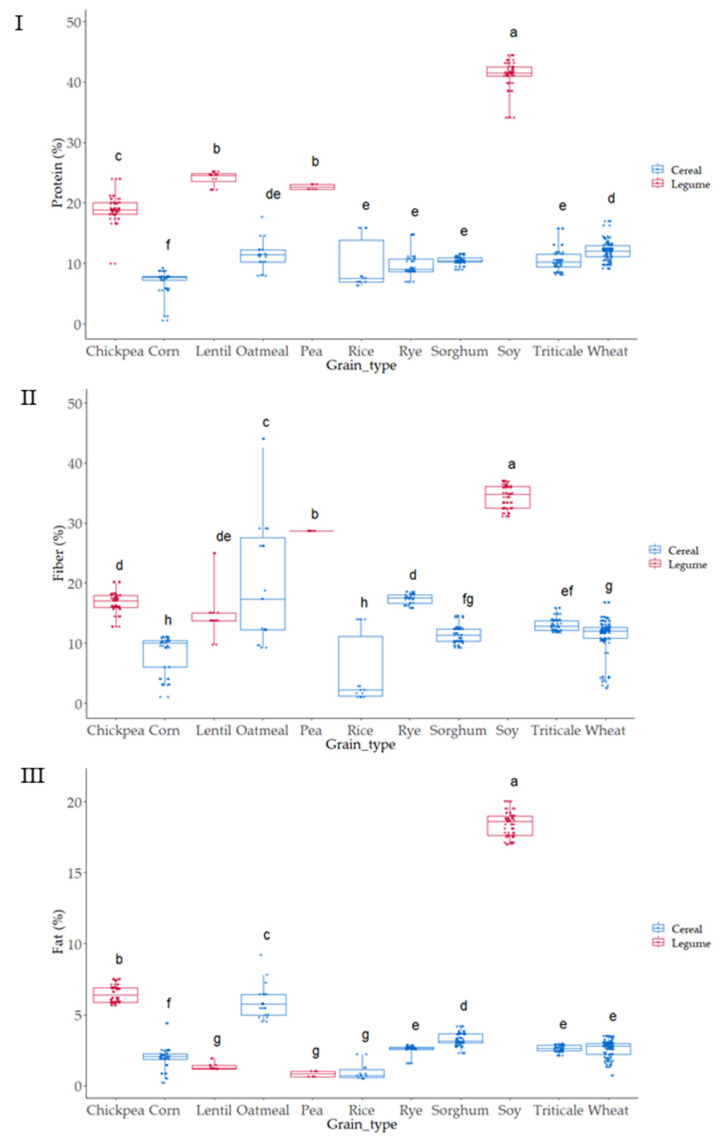
Boxplot distribution for (**I**) Protein, (**II**) Fat, and (**III**) Fiber (Total Dietary Fiber) according to the grain. Data was expressed in percentages (g 100 g^−1^ d.m.). Letters denote statistical differences between means (one-way ANOVA, posthoc Duncan’s test, *p* ≤ 0.05).

Proximal analysis (protein, fat, and fiber) was evaluated in all samples as it has been suggested that compositional variations can affect the antioxidant capacity of grains [29,30] (Figure 2 and Figure 3).

As expected, grain showed a wide variation in protein content; values ranged from 6 to 40 g 100 g^−1^ (Figure 2 and Figure 3I). The lowest values corresponded to cereals, corn and rice, where the lowest protein content appeared and pulses where the highest values were observed. These results are within the range of those reported by the USDA food database [31], which reports cereal protein values in the range of 6.69–7.5 g 100 g^−1^ for rice, 3.0–16.7 g 100 g^−1^ for wheat, 10.1–15.1 g 100 g^−1^ for oat, or 5.43–6.67 g 100 g^−1^ for corn. Pulses had higher protein content than cereals ranging from 14.50 to 23.97 g 100 g^−1^ for chickpea or 22.19 to 25.13 g 100 g^−1^ for lentil. As expected, soy was the grain with the highest protein content (34.06–44.38 g 100 g^−1^) (Figure 2 and Figure 3I). Cereals had higher differences in protein content associated with the variety factor, as compared to pulses. Rice was the grain where major variability due to variety was observed.

Protein content and profile differ with species, variety, genotype, and environmental and fertilization conditions, among other factors [32,33]. The amount of protein is quite important from a nutritional point of view, but the protein profile and the ratio of secondary structure are relevant regarding thermal stability after the process and in their later digestibility [34]. For example, grains with higher β-sheet content are easier to cook versus normal grains [35]. The reason is that temperature alters protein structure affecting disulphide and hydrogen linkages resulting in the reorganization of protein structure and becoming more or less accessible during protein digestibility. These changes can affect their antioxidant properties since many of the protein fragments produced during digestion exhibit antioxidant ability, as previously demonstrated in oats [36], rye [37], wheat [38], buckwheat, rice, corn [39], soybean, lentil, and chickpeas [40,41,42] among other grains.

Fat was also evaluated in all the grains (Figure 2 and Figure 3II), as variations in this fraction can affect the antioxidant status of cereals and pulses [43,44]. The content ranged from 2 to 5 g 100 g^−1^ in cereal, similar to previous results by other authors, which reported 1.9 g 100 g^−1^ for rice, 2.0 g 100 g^−1^ for wheat, 3.9 for g 100 g^−1^ corn, and 3.3 for g 100 g^−1^ sorghum [39]. Pulses such as lentil, pea, and chickpea showed content from 1 to 4 g 100 g^−1^ and soy showed, as it also occurred in protein, the highest values ranging from 31 to 38 g 100 g^−1^. Oatmeal, chickpea, and soy were the grains where the effect of variety produced a higher dispersion of results in fat content.

Total dietary fiber (TDF) has been strongly related to food antioxidant capacity [7,45]. TDF, according to the European Food Safety Authority (EFSA), contains nondigestible carbohydrates and lignin, nonstarch polysaccharides (cellulose, hemicelluloses, pectins, and hydrocolloids such as β-glucans), resistant oligosaccharides (fructo-oligosaccharides and galacto-oligosaccharides), other resistant oligosaccharides, and resistant starch (RS). The lowest values were observed in corn, which ranged from 3.49 to 10.85 g 100 g^−1^, and the highest in soy, with values from 31.60 to 37 g 100 g^−1^; the results agreed with TDF reported for grains [46]. The content in TDF was strongly affected by the variety studied, especially in the case of corn, oatmeal, and rice, where the variability associated was higher.

### 3.2. Colorimeter Analysis Characteristics of the Native Flours

**Figure 4:**
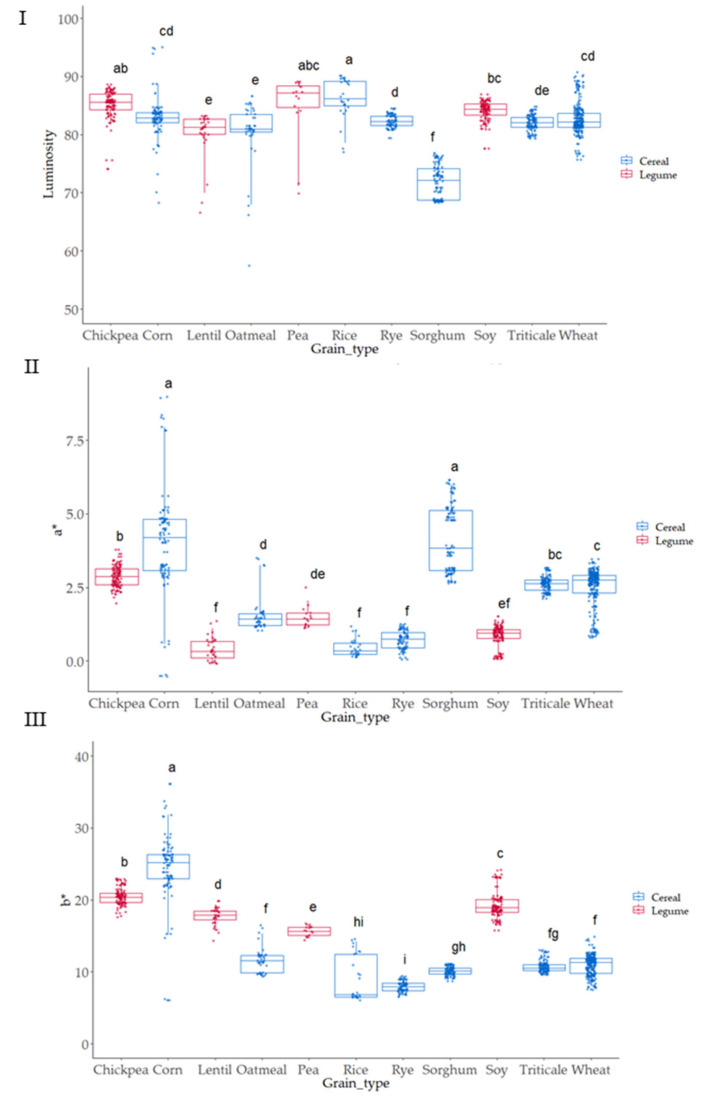
Boxplot distribution for (**I**) Luminosity, (**II**) a* and (**III**) b* according to the grain. Letters denote statistical differences between means (one-way ANOVA, posthoc Duncan’s test, *p* ≤ 0.05).

The color of the grain samples was evaluated, and CIELAB parameters (L*, a* and b*, Figure 4I–III) obtained. The color was measured since it is a quick assessment that can show a certain significant (*p* ≤ 0.05) association with antioxidant activity [47]. The type of grain was statistically significant (*p* ≤ 0.05) on L values; the darkest values were observed in sorghum; meanwhile, the highest lightness was observed in corn grains. The highest a* values (intensity of red) appeared in corn and sorghum samples, while the grains with lowest a* values (lowest intensity of red) were observed in lentil, rye, and soy. Significant differences (*p* ≤ 0.05) were observed when considering the b* parameter (blueness-yellowness). Positive b* values were observed in all samples indicating a yellow component in all the cases; the highest values were observed for corn, chickpea, and soy, and the lowest b* values (lowest yellowness) were obtained in rye, rice, and sorghum. The parameter a* was highly dispersed depending on the variety in the case of corn and sorghum; meanwhile, in b* value, corn and rice were the two grains that showed higher variability in yellowness as related to variety.

### 3.3. Total Phenol (TP) Content of the Native Flours

**Figure 5:**
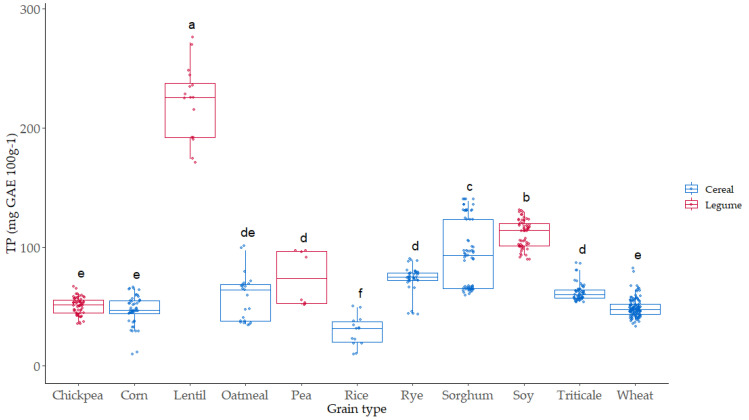
Boxplot distribution for TP (mg GAE 100 g^−1^) according to the grain. Data are mean values. Letters denote statistical differences between means (one-way ANOVA, posthoc Duncan’s test, *p* ≤ 0.05).

Phenolic compounds are secondary metabolites linked with the color and antioxidant activities of grains. The redox potentials of the phenolic compounds are the key factors for their antioxidant properties. Total phenol (TP) content was evaluated in all the samples (Figure 5). The values ranged from 25 mg GAE 100 g^−1^ in rice to 250 mg GAE 100 g^−1^ in lentil, where the highest values were observed.

Lentil is a legume with a high level of phenol compounds and high antioxidant activity, as it has been described previously by different authors [48,49]. Soy was the second grain with higher TP content (120 mg GAE 100 g^−1^), significantly higher than sorghum (90 mg GAE 100 g^−1^), which was the cereal with the highest TP content. The lowest TP values appeared in three of the cereals studied (rice, corn, and wheat) and chickpea. The effect of variety on TP was more significant in the case of sorghum, lentil, pea, and oatmeal. In the case of triticale, rye, wheat, corn, and chickpea, the TP content showed a lower dispersion associated with variety.

### 3.4. Oxygen Radical Absorbance Capacity (ORAC) of the Native Flours

**Figure 6:**
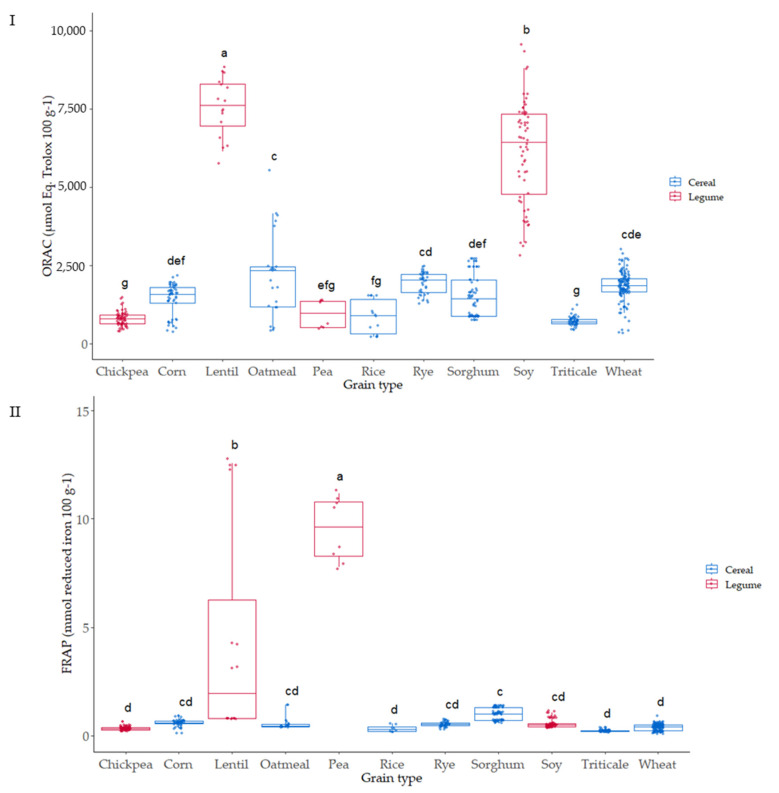
Box plot distribution for (**I**) ORAC (µmol Eq Trolox 100 g^−1^) and (**II**) FRAP (µmol reduced iron 100 g^−1^) according to the grain. Data are mean values. Letters denote statistical differences between means (one-way ANOVA, post hoc Duncan’s test, *p* ≤ 0.05).

The relevance of the ORAC methodology for antioxidant activity to in vivo conditions is based on the free radical source used (peroxyl), which shows the highest prevalence in human biology [50]. The ORAC assay was used to evaluate the ability of the 136 varieties of cereal and pulse grains studied to scavenge peroxyl free radicals; the values ranged from 437 to 7962 µmol Eq Trolox 100 g^−1^ dry weight (Figure 6I). The pulses, lentil and soy, showed the highest ORAC values compared to the rest of the grains analyzed. A significant effect of the variety was observed for the different grains, which can be associated with genetic and environmental factors, as has been observed in different studies [51]. The values observed agreed with hydrophilic ORAC values reported by USDA [52] for these types of grain.

### 3.5. Ferric-Reducing Antioxidant Power Assay (FRAP) of the Native Flours

Reducing power was also evaluated in all the samples (Figure 6II); FRAP ranged from 0.24 to 22.6 (µmol reduced iron 100 g^−1^). Pea was the grain where higher reducing power was observed, 15–20-fold the values of the other grains analyzed. Lentil also showed high FRAP capacity after pea, presenting the highest differences due to variety. In contrast, a very low variability due to variety was observed in most of the grains.

### 3.6. Parameter Correlations and Distribution of Parameters Based on Principal Component Analysis (PCA) and T-Distributed Stochastic Neighbor Embedding (T-SNE) of Native Flours

The degree of correlation between antioxidants (TP, ORAC, and FRAP), proximal parameters (proteins, fiber, and fat), and color parameters (L* a* and b*) was analyzed. (Supplementary Figure S1). Among the methods used to evaluate the antioxidant capacity of the samples, a higher correlation was found between ORAC and total phenol content (R^2^ 0.47). The highest correlation of composition parameters with antioxidant markers was for fiber and ORAC (R^2^ 0.49) and the lowest was for fat and ORAC (R^2^ 0.23). According to the results, ORAC was a feature that explained that TAC was associated not only with total phenol but also with other compounds involved directly or indirectly with antioxidant activity, such as protein (peptides) and fiber.

**Figure 7:**
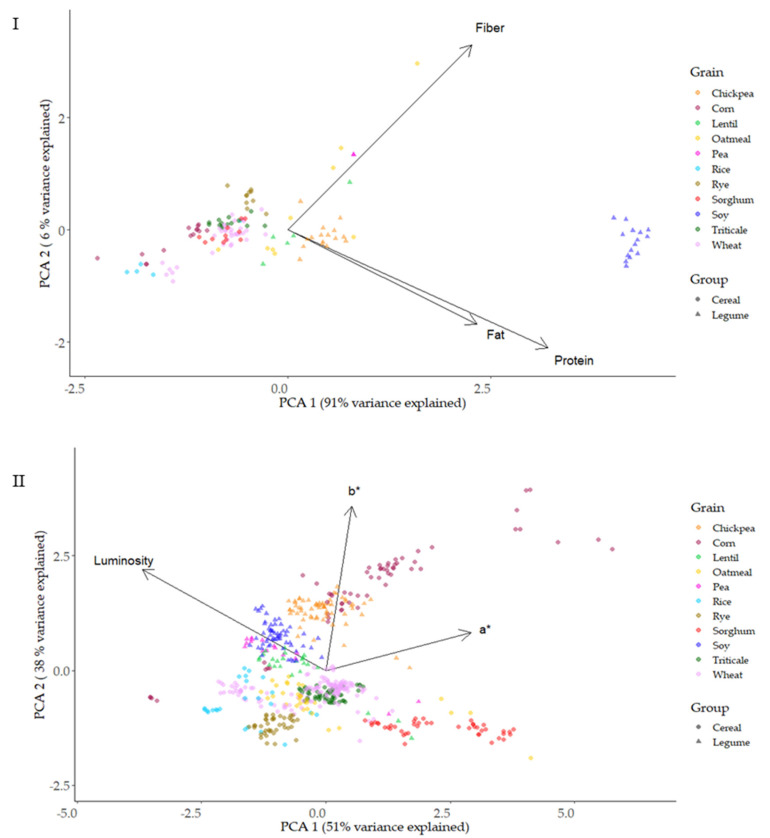
Representation of the proximal profile (**I**) and colorimeter parameters (**II**) of the grain types based on principal components analysis (PCA).

A projection of the proximal composition (protein, fiber, fat) and type of grain on the principal component plane, with PC1 and PC2 on the X and Y axes, respectively, are presented in Figure 7I. PC1 explained 91% of the variance; meanwhile, PC2 explained 6%. PC1 and PC2 are two distinct main clusters, cereals and pulses. Protein/fat and fiber showed an orthogonal spatial disposition, contributing to separate different type of grains. Fat resulted in redundancy with protein to identify the different types of grain. Protein appeared as the most useful parameter to segregate the different samples. Soy, pea, chickpea, and lentil appeared clearly separated from cereals, with the exception of oatmeal. Higher fat content was the proximal composition variable that clustered oatmeal within the pulses.

Figure 7II shows the PCA for color parameters L*a*b* and the type of grain. PC1 explained 51% of the variance, while PC2 only 38%. This level of variance explanation was lower than that found in the PCA of compositional parameters. A good separation of different samples was not achieved with the color parameters, as can be observed in the intermeshing of the two groups of grains (cereals and pulses) and the species studied.

**Figure 8:**
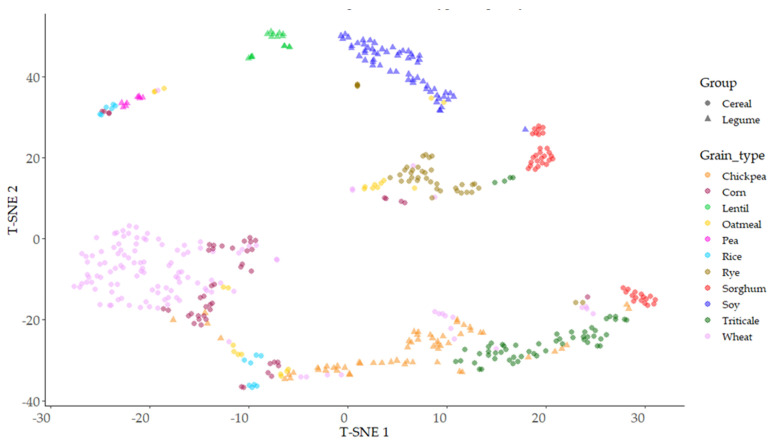
Distributed stochastic neighbor embedding (T-SNE) distribution of type of grains based on their antioxidant parameters (TP, ORAC, and FRAP).

With the objective of finding nonlinear similarities in the antioxidant capacity between the different grains, t-distributed stochastic neighbor embedding (T-SNE) analysis was used, with the aim of reducing initial spatial dimensions and representing the observed data based on the three antioxidant parameters evaluated. Different perplexity levels were used to find the highest separation between samples, 20 being the level of perplexity where better stratification was obtained (Figure 8). The legumes lentil and soy were the two grains showing single clusters of points, appearing with a relative similarity between them and clearly separated from the rest of the samples. The rest of the legumes (chickpea, pea) and all cereals were distributed in groups with different levels of fragmentation. Wheat showed a wide, round area of the main distribution, with some small groups of samples scattered along axis T-SNE1; wheat was clearly separated from the other cereals except corn. Rye distribution was clearly separated from wheat; these two cereals, wheat and rye, showed similar variation in relation to axes T-SNE1 and T-SNE2. The rest of the cereals showed fragmented dispersions, with projections separated in equivalent groups, as it occurred in rice, corn, oatmeal, and sorghum, or highly dependent on the T-SNE1 axis, as in the case of triticale.

### 3.7. Effect of Thermal Treatment on Total Antioxidant Capacity (TAC)

Baked goods require a thermal process prior to consumption; the thermal treatment conditions (time and temperature) will determine the final antioxidant capacity of the product through the degradation of thermally unstable polyphenol compounds and formation of the novo antioxidants from the polymerization of Maillard reaction products [53,54]. Baked good models (biscuits) were used for the kinetic study to evaluate the effect of temperature on the TAC. Wheat and rye were selected for model development and training, mainly due to their small variabilities and similar profiles in regard to model input variables (protein and fiber content), and also relatively small variability and similar profiles in antioxidant capacity results (silhouette coefficients for each grain type and antioxidant parameters are reported in Supplementary Table S1); regarding varieties, Berdun (wheat) and Loretto, Igor, and Teodor (rye) were selected due to their distribution of antioxidant data closer to a normal distribution compared with the rest of the varieties.

**Figure 9:**
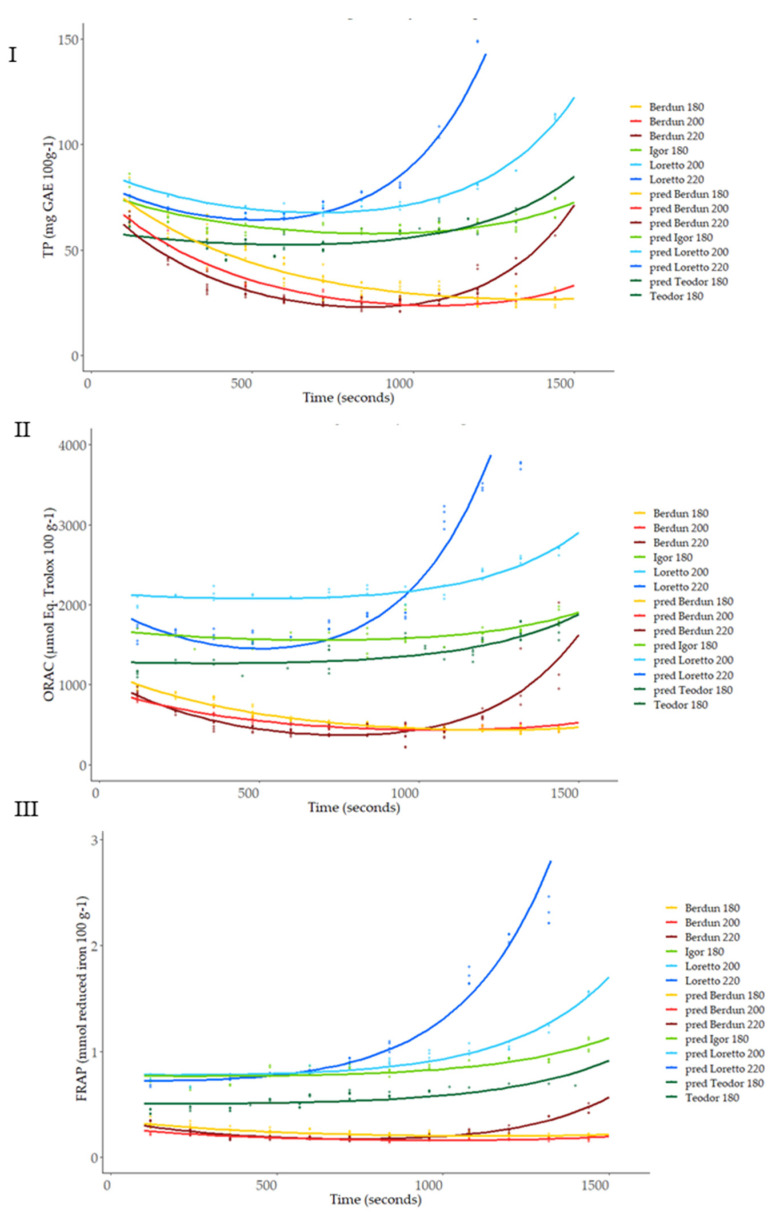
Level curves of total phenols, ORAC and FRAP, predicted with the first level model for the series according to baking temperature.

Figure 9 shows the total antioxidant capacity of model-baked products (biscuits) prepared using wheat and rye flours. TP content was affected by the baking thermal process (Figure 9I). An initial decrease in the TP was observed, regardless of the type of flour used (wheat/rye), with increasing temperatures until approximately 800 s.

After this time, an increase in TP is observed in wheat biscuits baked at 220 °C and in rye biscuits at 180, 200, and 220 °C. At the end of the baking process (1500 s), wheat TP values were similar to or lower than the initial values, while significant increases after thermal processing were observed in the case of rye samples treated at 200 and 220 °C. Rye samples treated at 180 °C did not show significant variation after the baking process. TP methodology based on the Folin−Ciocalteu reagent can reflect not only phenolic content but also the presence of other antioxidants [11]. Rye TP content showed lower sensitivity to temperature compared to wheat. This behavior could be explained by a faster degradation of thermolabile antioxidants, in the case of wheat, or a significantly higher formation of Maillard reaction products (MRPs), which in the case of rye, would be favored by its higher content in lysine compared to wheat [55]. Previously, a correlation between acrylamide formation and an increment in antioxidant capacity in wheat and rye bread models has been suggested [56], suggesting health-related benefits with low-temperature, long-time treatments.

On the other hand, degradation of acrylamide may also occur in high-temperature treatments [57], while final MRPs, melanoidins, have shown biological activities and could be considered as antioxidant fiber [57,58].

Similar behavior of the antioxidant capacity measured against peroxyl radicals (ORAC) was observed compared to TP results (Figure 9II). The antioxidant capacity of wheat samples decreased in the initial stages of the thermal treatment. After 1000 s of treatment, the antioxidant capacity of all samples (wheat and rye) treated at higher temperatures (200 °C and 220 °C) increased and, in the case of the rye samples, the levels reached (2600–3800 µmol Eq Trolox 100 g^−1^) were significantly higher than the initial levels (1500–2000 µmol Eq Trolox 100 g^−1^). The gradual formation during the thermal process of high molecular weight melanoidins and binding of low molecular weight MRPs to melanoidin skeletons can contribute to the final antioxidant activity of the samples. These compounds react with the peroxyl radicals and are measured by the ORAC assay, as happened in the case of the Folin−Ciocalteu reagent in the TP method [59].

The ferric-reducing ability (FRAP values) of the rye was significantly higher than that of the wheat (Figure 9III). As observed in the TP and ORAC assays, a decrease in FRAP values was observed for wheat samples up to 800 s of treatment. After this period, the FRAP values increased in a temperature-dependent manner. Samples treated at 220 °C showed the highest final ferric-reducing values (FRAP) values. This behavior again could be associated with the presence of melanoidins, which have been reported among their biological activities’ significant ferric-reducing capacity [54].

### 3.8. Modelling of the Effect of Thermal Treatment on Total Antioxidant Capacity (TAC)

Previous to model building, experimental data (Figure 9) were assumed to follow a function with two exponential parts: a negative exponential simulating antioxidant compound degradation and a positive exponential simulating de novo synthesis of antioxidant compounds (Equation (1), ‘*t*’ represents time, ‘*T*’ temperature and $\hat{Y}$ antioxidant capacity as TP, ORAC, or FRAP).

(1) $$\hat{Y}=ϴ+\alpha_{1}\cdot \;e^{-\beta_{1}*t*T}+\frac{\alpha_{2}}{100}\;\cdot \;e^{\beta_{2}*t*T}$$

As a first exploratory search of space of hyperparameters (*θ*, *α*_1_, *α*_2_, *β*_1_, *β*_2_), iterative optimization tools were used (R packages “nlme” and “nls”). Parameters *β*_1_ and *β*_2_ were fixed at 0.08 and 0.12, respectively, due to their low influence on curve shape. The number of hyperparameters was reduced to three (theta, alpha1 and alpha2), simplifying the solution search space and facilitating convergence in the search for an optimal solution (Equation (2)).

(2) $$\hat{Y}=ϴ+\alpha_{1}\cdot e^{-0.08*t*T}+\frac{\alpha_{2}}{100}\;\cdot \;e^{0.12*t*T}$$

#### 3.8.1. First Level of Modeling

As each of the seven available curves could be clustered based on factors type of grain and temperature, a hierarchical model was selected. A Bayesian hierarchical model was used to determine the values of theta, alpha1, and alpha2 parameters for each of seven combinations of temperature and variety, which uses the baking temperature, time, and type of grain as predictors. After that, the median value of Monte Carlo distributions obtained in the Bayesian training for each configuration (variety, temperature) was obtained (Table 1), and training datasets for the SVM model for prediction of theta, alpha1, and alpha2 are shown in Supplementary Tables S2–S4. Values corresponding to the main parameters of the ORAC and FRAP models are displayed in Supplementary Tables S6 and S7, respectively.

**Figure 10:**
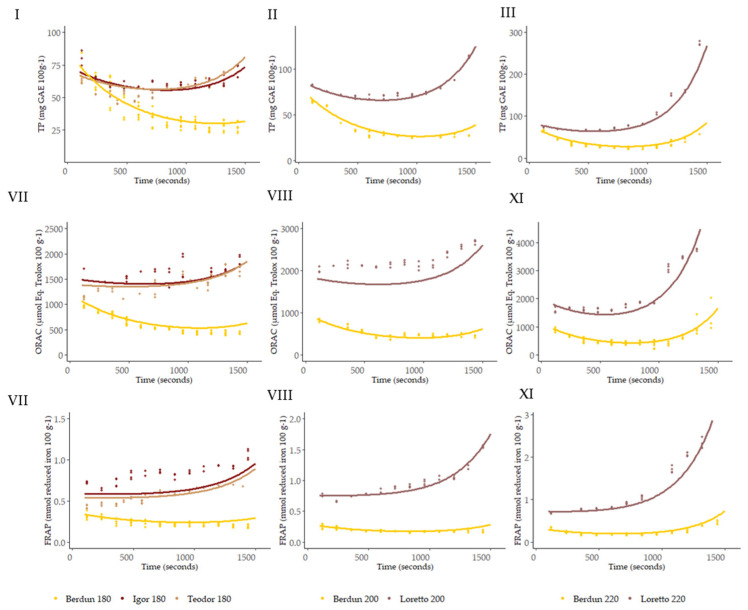
Curves of the level of TP (**I**–**III**), ORAC (**IV**–**VI**), and FRAP (**VII**–**IX**) predicted with the second level models for the series according to baking temperature.

With the use of the Bayesian hierarchical model, the parameters (theta, alpha1, and alpha2) of the double exponential curve were obtained; the best-fitting curves obtained are shown in Figure 10.

#### 3.8.2. Second Level of Modeling

Using as reference the optimal parameters (theta, alpha1, and alpha2) fitted with the Bayesian hierarchical model, a training procedure was carried out using as input data the compositional (protein and fiber), type of grain (wheat or rye) and temperature (180, 200 or 220 °C). Independent training steps using support vector regression (SVM) for each combination of parameters (theta, alpha1, and alpha2) and antioxidant value (TP, ORAC, and FRAP) were carried out. Datasets used for training in the different models are provided in Supplementary Tables S1–S3. The corresponding cost parameter and R^2^ obtained for SVM model training can be found in Supplementary Table S4.

Using the obtained SVM models, it is possible to predict the antioxidant capacity (TP, ORAC, and FRAP) at any given thermal processing temperature and time, protein, and fiber as proximal parameters, and wheat or rye as types of grain (Figure 10).

Therefore, relying solely on the secondary models to determine the shape of the curve to be predicted, that is, using baking temperature, grain type, protein, and fiber as predictors, the fit shown below was achieved (Table 2, Figure 10, and Supplementary Figure S2), where it should be noted that they are accompanied by graphs in which the values of the residuals are represented and that, except for the predictions of Teodor and Igor at 180 °C, all the models manage to explain more than 80% of the variance present in the data.

#### 3.8.3. Sensitivity of Modeling

**Figure 11:**
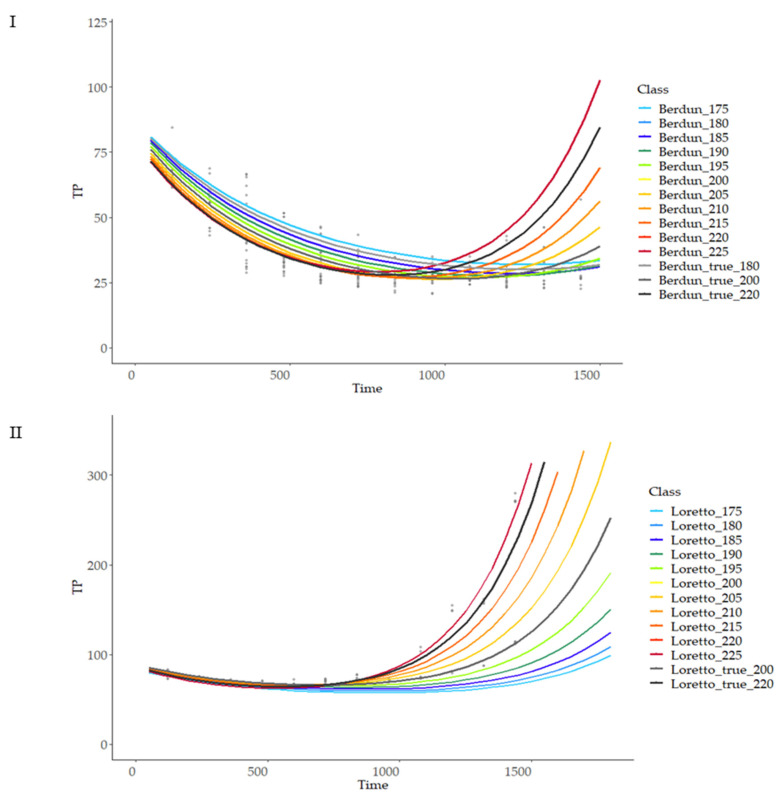
Prediction curves for total phenol content (TP) based on different baking temperatures on wheat (**I**) and rye (**II**).

To estimate model stability, the variability of the predictions after different entry values was analyzed. Temperature and proximal composition (protein, fiber) independent variations (maintaining the rest of variables fixed) were used as inputs for the model and compared with model outcome using available values of temperature and composition as reference. Figure 11 shows the predicted behaviour of rye and wheat at a range of temperatures from 175 to 225 °C at 5 °C intervals.

**Figure 12:**
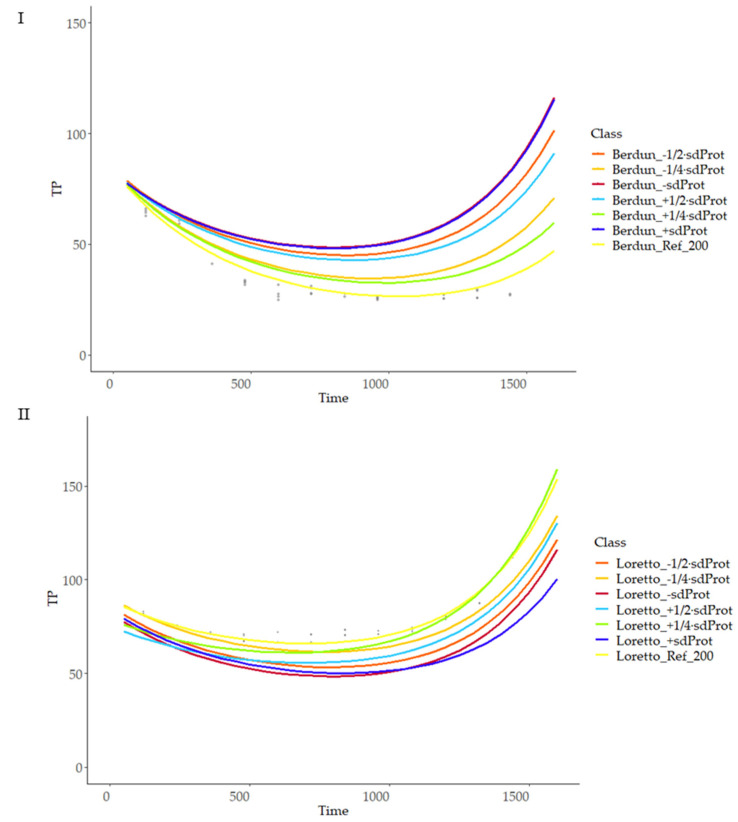
Prediction curves for total phenol content (TP) based on different protein content (g 100 g^−1^) on wheat (**I**) and rye (**II**).

**Figure 13:**
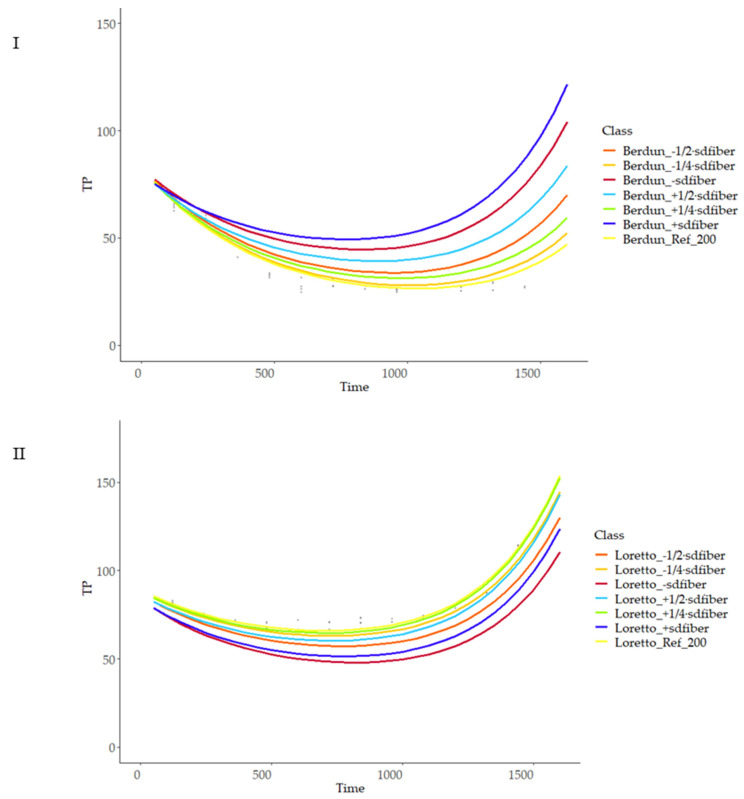
Prediction curves for total phenol content (TP) based on different fiber (g 100 g^−1^) content on wheat (**I**) and rye (**II**).

As can be observed, the model is more sensitive to temperature changes at advanced treatment times, with a linear relationship of temperature and curve slope, with lower variation at the initial stages of the thermal process; there was a higher variability of the final TP values in the case of rye products compared with wheat (Figure 11), revealing more critical importance in the first case of the temperature compared with the second, when selecting a treatment temperature with the objective of obtaining a certain TP final value. Similarly, more sensitivity of the model was observed in the case of rye compared to wheat when studying the model sensitivity to protein and fiber content variations (Figure 12 and Figure 13).

The total antioxidant capacity profiles of a range of cereal and legume flours, as evaluated through three different methods (TP, ORAC, and FRAP), and their distribution variability related to proximal composition and color were reported. The distribution of the variability of the antioxidant data was significantly affected by the protein and fiber composition of the samples. The variability of legume flours showed in the generally higher values of antioxidant capacity compared to cereals, with special relevance to lentil flour. Reduced dimensionality techniques (PCA and T-SNE) applied to the data showed wheat and rye as two samples with homogeneous distribution and well separated.

**Figure 14:**
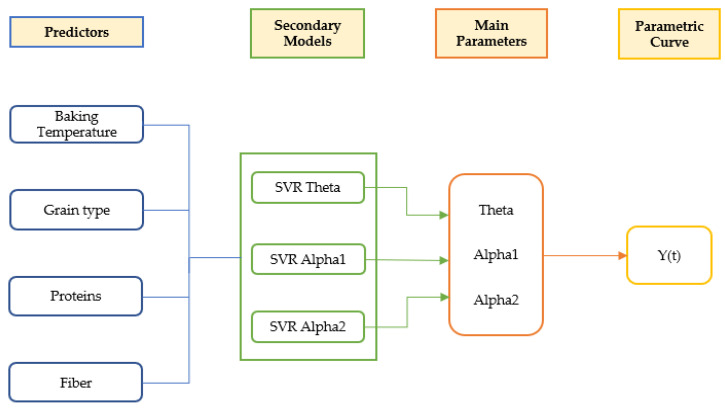
Schematic representation of the global model.

The behavior of the antioxidant capacity profiles of the baked products suffered a decrease over the first half of the treatment times, with a subsequent increase afterward. A predictive model for the estimation of the TAC of baked products, using Bayesian and SVM modelling tools and considering protein and fiber content, was developed. A double exponential parameterized curve that reflects the initial decrease and subsequent increase as a consequence of the processes of degradation and generation of the antioxidant compounds is proposed. An estimation of the antioxidant capacity of a baked product during its thermal process has been achieved; this model (Figure 14) is intended to be improved with further types of grains and varieties to create a tool that may be of interest to the food industry; this tool would estimate optimal processing conditions in order to maximize the antioxidant capacity of baked products, using as input values type of grain, protein, and fiber content, and temperature and time of the process.

The use of this type of tool may provide more accurate information to the agrifood industry, reducing the use of artificial antioxidants while enhancing naturally present antioxidant compounds. In this way, this research may serve to boost the development of innovative products relying on cleaner labels and antioxidant properties as potential claims for consumers.

## Figures and Tables

**Table 1:** Values corresponding to the parameters theta, alpha1, and alpha2 of TP, ORAC, and FRAP models.

Setting		TP			ORAC			FRAP	
	Theta	Alpha1	Alpha2	Theta	Alpha1	Alpha2	Theta	Alpha1	Alpha2
Berdun 180 °C	0.605	1.985	0.100	0.291	0.826	0.117	0.296	0.297	0.059
Igor 180 °C	1.497	0.850	0.573	0.317	0.588	0.106	0.231	0.246	0.061
Teodor 180 °C	1.391	0.375	1.013	0.112	0.926	0.566	0.186	0.409	0.292
Berdun 200 °C	0.431	1.960	0.265	1.875	0.134	0.564	1.285	0.045	1.020
Loretto 200 °C	1.717	0.938	1.022	0.873	1.016	3.034	1.131	0.090	2.658
Berdun 220 °C	0.309	1.976	0.566	1.371	0.220	0.450	1.278	0.034	0.680
Loretto 220 °C	1.462	1.020	2.516	1.140	0.062	0.686	0.836	0.025	0.755

**Table 2:** R^2^ of SRV models.

Temperature	Variety	R^2^_TP	R^2^_ORAC	R^2^_FRAP
180 °C	Berdun	0.807	0.655	−0.372
180 °C	Teodor	0.021	0.500	0.387
180 °C	Igor	0.396	−0.404	−2.081
200 °C	Berdun	0.893	0.716	−0.266
200 °C	Loretto	0.952	−2.051	0.935
220 °C	Berdun	0.632	0.712	0.423
220 °C	Loretto	0.919	0.844	0.881

## Data Availability

Data is contained within the article or Supplementary Materials.

## Supplementary Materials

The following supporting information can be downloaded at: https://www.mdpi.com/article/10.3390/foods12173208/s1.

## References

[1] Sajdakowska M., Gębski J., Jeżewska-Zychowicz M., Królak M. (2021). Consumer Choices in the Bread Market: The Importance of Fiber in Consumer Decisions. Nutrients.

[2] Anderson J., Hanna T. (1999). Whole grains and protection against coronary heart disease: What are the active components and mechanisms?. Am. J. Clin. Nutr..

[3] Anderson J.W., Hanna T.J., Peng X., Kryscio R.J. (2000). Whole grain foods and heart disease risk. J. Am. Coll. Nutr..

[4] Bourdon I., Yokoyama W., Davis P., Hudson C., Backus R., Richter D., Knuckles B., Schneeman B.O. (1999). Postprandial lipid, glucose, insulin and cholecystokinin responses in men fed barley pasta enriched with beta-glucan. Am. J. Clin. Nutr..

[5] Slavin J., Jacobs D., Marquart L. (1997). Whole-grain consumption and chronic disease: Protective mechanisms. Nutr. Cancer.

[6] Martín-Diana A.B., García-Casas M.J., Martínez-Villaluenga C., Frías J., Peñas E., Rico D. (2021). Wheat and Oat Brans as Sources of Polyphenol Compounds for Development of Antioxidant Nutraceutical Ingredients. Foods.

[7] Călinoiu L.F., Vodnar D.C. (2018). Whole Grains and Phenolic Acids: A Review on Bioactivity, Functionality, Health Benefits and Bioavailability. Nutrients.

[8] Europe Phenolic Antioxidant Market Insights Forecasts to 2032. https://www.sphericalinsights.com/reports/europe-phenolic-antioxidant-market.

[9] Pruteanu L.L., Bailey D.S., Grădinaru A.C., Jäntschi L. (2023). The Biochemistry and Effectiveness of Antioxidants in Food, Fruits, and Marine Algae. Antioxidants.

[10] Pellegrini N., Vitaglione P., Granato D., Fogliano V. (2020). Twenty-five years of total antioxidant capacity measurement of foods and biological fluids: Merits and limitations. J. Sci. Food Agric..

[11] Huang D., Ou B., Prior R.L. (2005). The chemistry behind antioxidant capacity assays. J. Agric. Food Chem..

[12] Huang D., Ou B., Hampsch-Woodill M., Flanagan J.A., Deemer E.K. (2002). Development and validation of oxygen radical absorbance capacity assay for lipophilic antioxidants using randomly methylated cyclodextrin as the solubility enhancer. J. Agric. Food Chem..

[13] Niki E. (2009). Lipid peroxidation: Physiological levels and dual biological effects. Free Radic. Biol. Med..

[14] Vanegas S.M., Meydani M., Barnett J.B., Goldin B., Kane A., Rasmussen H., Brown C., Vangay P., Knights D., Jonnalagadda S. (2017). Substituting whole grains for refined grains in a 6-wk randomised trial has a modest effect on gut microbiota and immune and inflammatory markers of healthy adults. Am. J. Clin. Nutr..

[15] Björck I., Östman E., Kristensen M., Mateo Anson N., Price R.K., Haenen G.R.M.M., Havenaar R., Bach Knudsen K.E., Frid A., Mykkänen H. (2012). Cereal grains for nutrition and health benefits: Overview of results from in vitro, animal and human studies in the HEALTHGRAIN project. Trends Food Sci. Technol..

[16] García-Castro A., Román-Gutiérrez A.D., Castañeda-Ovando A., Cariño-Cortés R., Acevedo-Sandoval O.A., López-Perea P., Guzmán-Ortiz F.A. (2022). Cereals as a Source of Bioactive Compounds with Anti-Hypertensive Activity and Their Intake in Times of COVID-19. Foods.

[17] Prior R.L., Wu X., Schaich K. (2005). Standardized methods for the determination of antioxidant capacity and phenolics in foods and dietary supplements. J. Agric. Food Chem..

[18] Tsiaka T., Kritsi E., Tsiantas K., Christodoulou P., Sinanoglou V.J., Zoumpoulakis P. (2022). Design and Development of Novel Nutraceuticals: Current Trends and Methodologies. Nutraceuticals.

[19] Halvorsen B.L., Holte K., Myhrstad M.C., Barikmo I., Hvattum E., Remberg S.F., Wold A.B., Haffner K., Baugerød H., Andersen L.F. (2002). A systematic screening of total antioxidants in dietary plants. J. Nutr..

[20] Halvorsen B.L., Carlsen M.H., Phillips K.M., Bøhn S.K., Holte K., Jacobs D.R., Blomhoff R. (2006). Content of redox-active compounds (ie, antioxidants) in foods consumed in the United States. Am. J. Clin. Nutr..

[21] Haytowitz D.B., Bhagwat S. (2010). USDA Database for the Oxygen Radical Absorbance Capacity (ORAC) of Selected Foods, Release 2.

[22] Takebayashi J., Oki T., Watanabe J., Yamasaki K., Chen J., Sato-Furukawa M., Tsubota-Utsugi M., Taku K., Goto K., Matsumoto T. (2013). Hydrophilic antioxidant capacities of vegetables and fruits commonly consumed in Japan and estimated average daily intake of hydrophilic antioxidants from these foods. J. Food Comp. Anal..

[23] Li Z., Nie K., Wang Z. (2016). Quantitative Structure Activity Relationship Models for the Antioxidant Activity of Polysaccharides.

[24] Borah P., Gupta D. (2017). Review: Support Vector Machines in Pattern Recognition. Int. J. Eng. Technol..

[25] AOAC (2005). Methods 990.03, 2003.05, 985.29 & 923.03. Official Methods of Analysis of AOAC International.

[26] Slinkard K., Singleton V.L. (1977). Total phenol analyses: Automation and comparison with manual methods. Am. J. Enol. Viticult.

[27] Ou B., Hampsch-Woodill M., Prior R.L. (2001). Development and validation of an improved oxygen radical absorbance capacity assay using fluorescein as the fluorescent probe. J. Agric. Food Chem..

[28] Benzie I.F.F., Strain J.J. (1996). The Ferric Reducing Ability of Plasma (FRAP) as measure of “antioxidant power”: The FRAP assay. Anal. Biochem..

[29] Badarinath A.V., Rao K.M., Chetty C.M.S., Ramkanth S., Rajan T.V.S. (2010). Review on In-vitro Antioxidant Methods: Comparisions, Correlations and Considerations. Inter. J. Pharm. Tech. Res..

[30] Nile S.H., Khobragade C.N., Park S.W. (2012). Optimized and Comparative Antioxidant Assays and Its Applications in Herbal and Synthetic Drug Analysis as an Antioxidants. Mini-Rev. Med. Chem..

[31] USDA (U.S. DEPARTMENT OF AGRICULTURE) Food Data Central. https://fdc.nal.usda.gov/.

[32] Singh N. (2017). Pulses: An overview. J. Food Sci. Technol..

[33] Singh B., Singh J.P., Shevkani K., Singh N., Kaur A. (2017). Bioactive constituents in pulses and their health benefits. J. Food Sci. Technol..

[34] Gulati P., Li A., Holding D., Santra D., Zhang Y., Rose D.J. (2017). Heating Reduces Proso Millet Protein Digestibility via Formation of Hydrophobic Aggregates. J. Agric. Food Chem..

[35] Parmar N., Singh N., Kaur A., Virdi A.S., Shevkani K. (2017). Protein and microstructure evaluation of harder-to-cook and easy-to-cook grains from different kidney bean accessions. LWT-Food Sci. Technol..

[36] Du Y., Esfandi R., Willmore W.G., Tsopmo A. (2016). Antioxidant activity of oat proteins derived peptides in stressed hepatic HepG2 cells. Antioxidants.

[37] Leung R., Venus C., Zeng T., Tsopmo A. (2018). Structure-function relationships of hydroxyl radical scavenging and chromium-VI reducing cysteine-tripeptides derived from rye secalin. Food Chem..

[38] Chen S., Lin D., Gao Y., Cao X., Shen X. (2017). A novel antioxidant peptide derived from wheat germ prevents high glucose-induced oxidative stress in vascular smooth muscle cells in vitro. Food Funct..

[39] Das A., Raychaudhuri U., Chakraborty R. (2012). Cereal based functional food of Indian subcontinent: A review. J. Food Sci. Technol..

[40] Torres-Fuentes C., Contreras M., Recio I., Alaiz M., Vioque J. (2015). Identification and characterization of antioxidant peptides from chickpea protein hydrolysates. Food Chem..

[41] Xie J., Du M., Shen M., Wu T., Lin L. (2019). Physico-chemical properties, antioxidant activities and angiotensin-I converting enzyme inhibitory of protein hydrolysates from Mung bean (Vigna radiate). Food Chem..

[42] Zhang X., He H., Xiang J., Li B., Zhao M., Hou T. (2021). Selenium-containing soybean antioxidant peptides: Preparation and comprehensive comparison of different selenium supplements. Food Chem..

[43] Konopka I., Grabiński J., Skrajda M., Dąbrowski G., Tańska M., Podolska G. (2017). Variation of wheat grain lipid fraction and its antioxidative status under the impact of delayed sowing. J. Cereal Sci..

[44] Ciudad-Mulero M., Matallana-González M.C., Cámara M., Fernández-Ruiz V., Morales P. (2020). Antioxidant Phytochemicals in Pulses and their Relation to Human Health: A Review. Curr. Pharm. Des..

[45] Núñez-Gómez V., González-Barrio R., Periago M.J. (2023). Interaction between Dietary Fiber and Bioactive Compounds in Plant By-Products: Impact on Bioaccessibility and Bioavailability. Antioxidants.

[46] Tosh S.M., Farnworth E.R., Brummer Y., Duncan A.M., Wright A.J., Boye J.I. (2013). Marcotte, M.; Benali, M. Nutritional Profile and Carbohydrate Characterization of Spray-Dried Lentil, Pea and Chickpea Ingredients. Foods.

[47] Muzolf-Panek M., Wa’skiewicz A. (2022). Relationship between Phenolic Compounds, Antioxidant Activity and Color Parameters of Red Table Grape Skins Using Linear Ordering Analysis. Appl. Sci..

[48] Rico D., Peñas E., Del Carmen García M., Rai D.K., Martínez-Villaluenga C., Frias J., Martín-Diana A.B. (2021). Development of Antioxidant and Nutritious Lentil (*Lens culinaris*) Flour Using Controlled Optimized Germination as a Bioprocess. Foods.

[49] Aguilera Y., Dueñas M., Estrella I., Hernández T., Benitez V., Esteban R.M., Martín-Cabrejas M.A. (2010). Evaluation of Phenolic Profile and Antioxidant Properties of Pardina Lentil as Affected by Industrial Dehydration. J. Agric. Food Chem..

[50] Prior R.L. (2015). Oxygen radical absorbance capacity (ORAC): New horizons in relating dietary antioxidants/bioactives and health benefits. J. Funct. Foods.

[51] Mpofu A., Sapirstein H.D., Beta T. (2006). Genotype and environmental variation in phenolic content, phenolic acid composition, and antioxidant activity of hard spring wheat. J. Agric. Food Chem..

[52] http://www.ars.usda.gov/nutrientdata.

[53] Blanch G.P., Ruiz del Castillo M.L. (2021). Effect of Baking Temperature on the Phenolic Content and Antioxidant Activity of Black Corn (*Zea mays* L.) Bread. Foods.

[54] Delgado-Andrade C., Rufián-Henares J.A., Morales F.J. (2005). Assessing the Antioxidant Activity of Melanoidins from Coffee Brews by Different Antioxidant Methods. J. Agric. Food Chem..

[55] Rani M., Singh G., Siddiqi R.A., Gill B.S., Sogi D.S., Bhat M.A. (2021). Comparative Quality Evaluation of Physicochemical, Technological, and Protein Profiling of Wheat, Rye, and Barley Cereals. Front. Nutr..

[56] Przygodzka M., Piskula M.K., Kukurová K., Ciesarová Z., Bednarikova A., Zieliński H. (2015). Factors influencing acrylamide formation in rye, wheat and spelt breads. J. Cereal Sci..

[57] Çelik E.E., Gökmen V. (2020). Formation of Maillard reaction products in bread crust-like model system made of different whole cereal flours. Eur. Food Res. Technol..

[58] Saura-Calixto F. (1998). Antioxidant dietary fiber product: A new concept and a potential food ingredient. J. Agric. Food Chem..

[59] Liu J., Gan J., Yu Y., Zhu S., Yin L., Cheng Y. (2016). Effect of laboratory-scale decoction on the antioxidative activity of Zhenjiang Aromatic Vinegar: The contribution of melanoidins. J. Funct. Foods.

