# Supplementary material for: Study of the Total Antioxidant Capacity (TAC) in Native Cereal−Pulse Flours and the Influence of the Baking Process on TAC Using a Combined Bayesian and Support Vector Machine Modeling Approach
Source: Foods. 2023 Aug 25;12(17):3208. doi: 10.3390/foods12173208 (PMC10487251; doi:10.3390/foods12173208)
Supplement: Supplementary file 1 [file foods-12-03208-s001.zip › foods-2521574-supplementary.pdf]

Figure 1. Biscuits before and after thermal treatment (baking process) at different temperatures (180, 200 and 220 °C and times (0-1500 s).

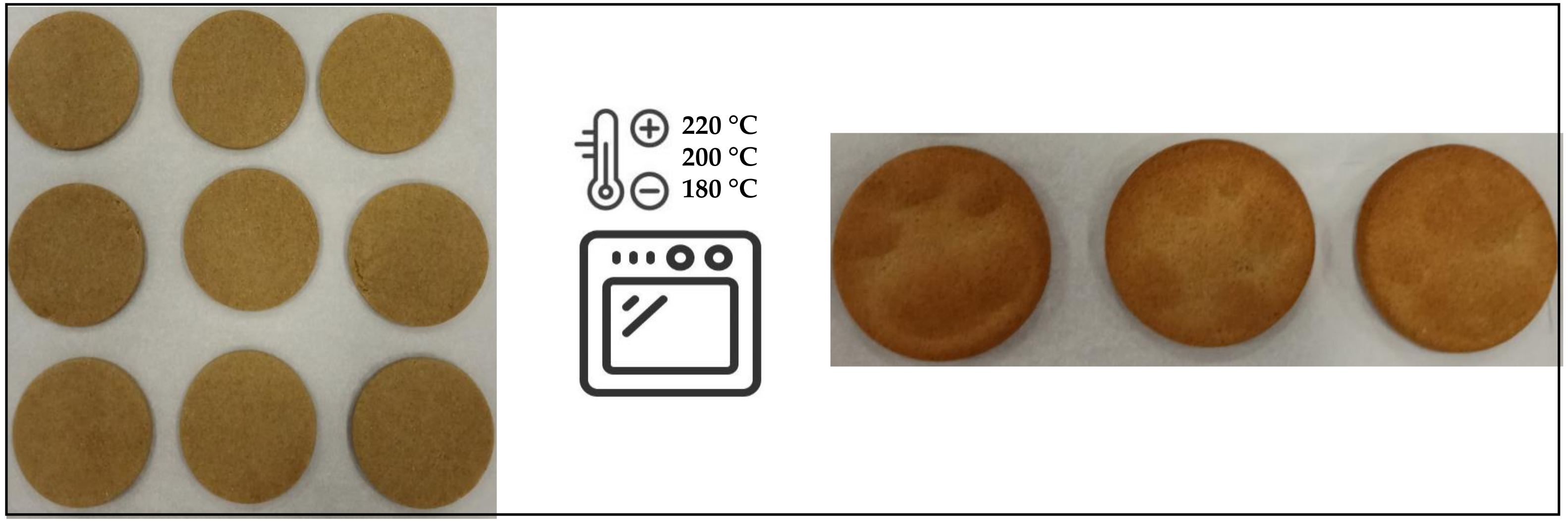

Figure 2. Stacked bar graph representing the proximal profile according to grain type. Letters of each colour denotes statistical differences between means (one-way ANOVA, post hoc Duncan's test, p ≤ 0.05). Data was express in percentage (g 100 g-1 d.m.).

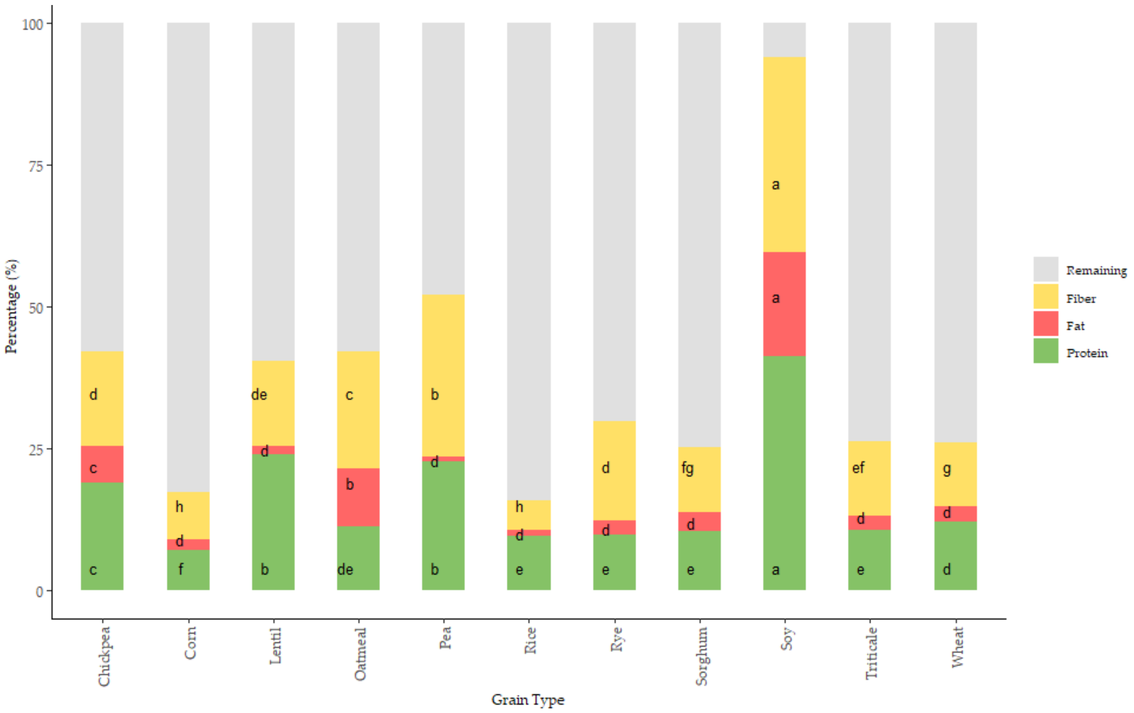

Figure 3. Box-Plot distribution for (I) Protein, (II) Fat and (III) Total Dietary Fiber (%) according to the grain. Data are mean values. Letters denotes statistical differences between means (one-way ANOVA, post hoc Duncan's test, p ≤ 0.05).

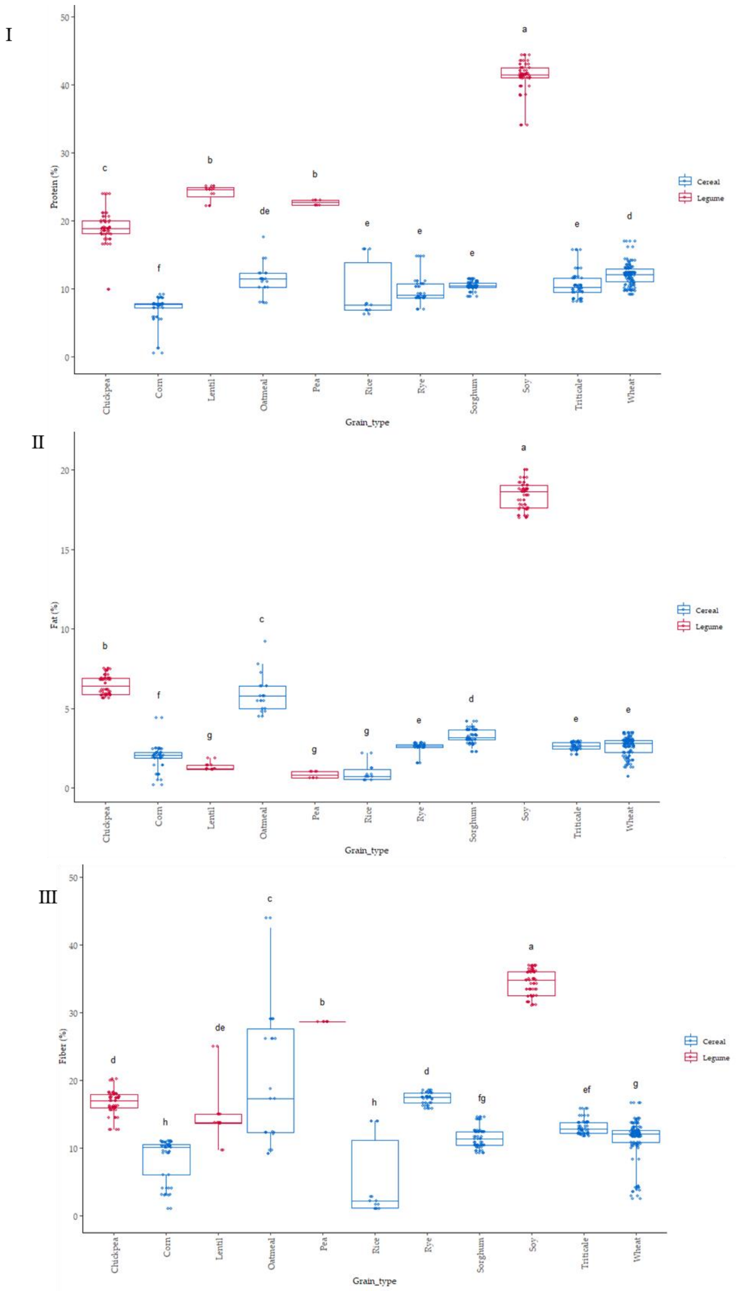

Figure 4. Box-Plot distribution for (I) Luminosity, (II) a* and (III) b* according to the grain. Data are mean values. Letters denotes statistical differences between means (one-way ANOVA, post hoc Duncan's test, p ≤ 0.05).

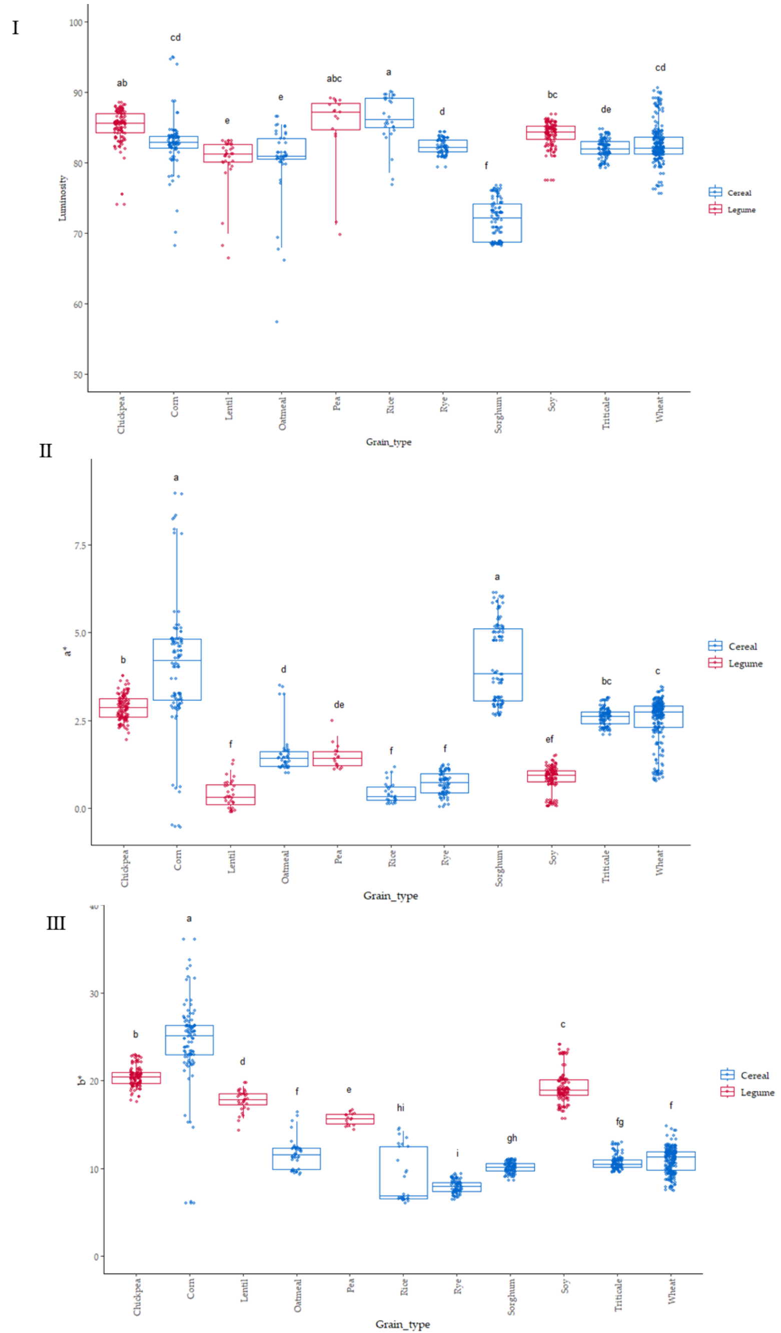

Figure 5. Box-Plot distribution for TP (mg GAE 100 g-1) according to the grain. Data are mean values. Letters denotes statistical differences between means (one-way ANOVA, post hoc Duncan's test, $p \leq 0.05$).

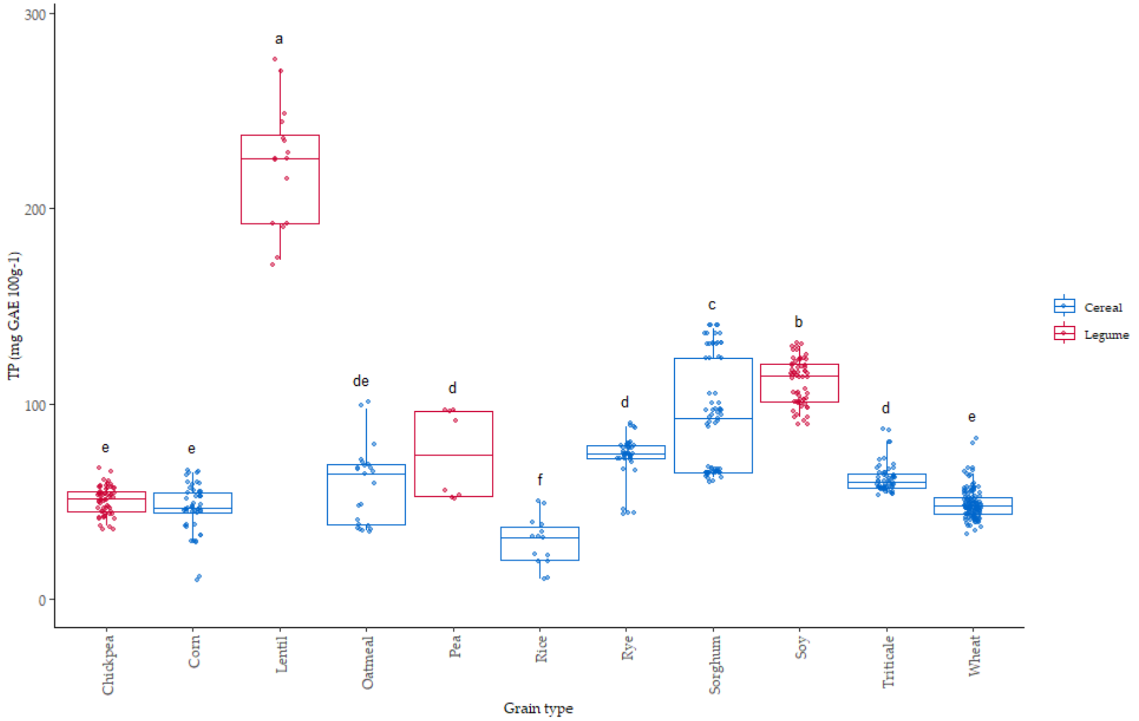

Figure 6. Box-Plot distribution for (I) ORAC (µmol Eq. Trolox 100 g-1) and (II) FRAP (µmol reduced iron 100 g-1) according to the grain. Data are mean values. Letters denotes statistical differences between means (one-way ANOVA, post hoc Duncan's test, p ≤ 0.05).

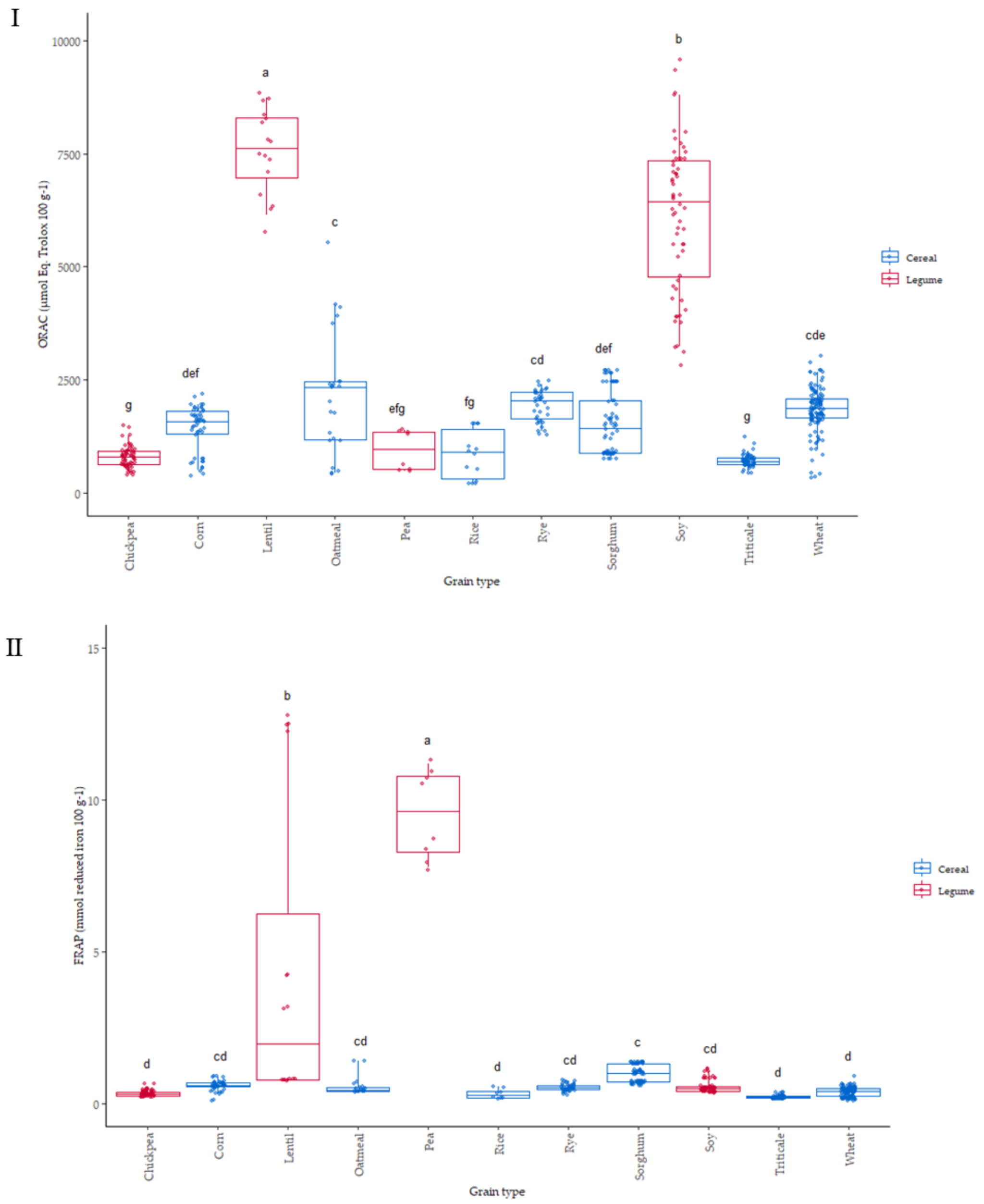

Figure 7. Representation of the chemical profile (I) and colourimeter parameters (II) of the grain types based on principal components analysis (PCA).

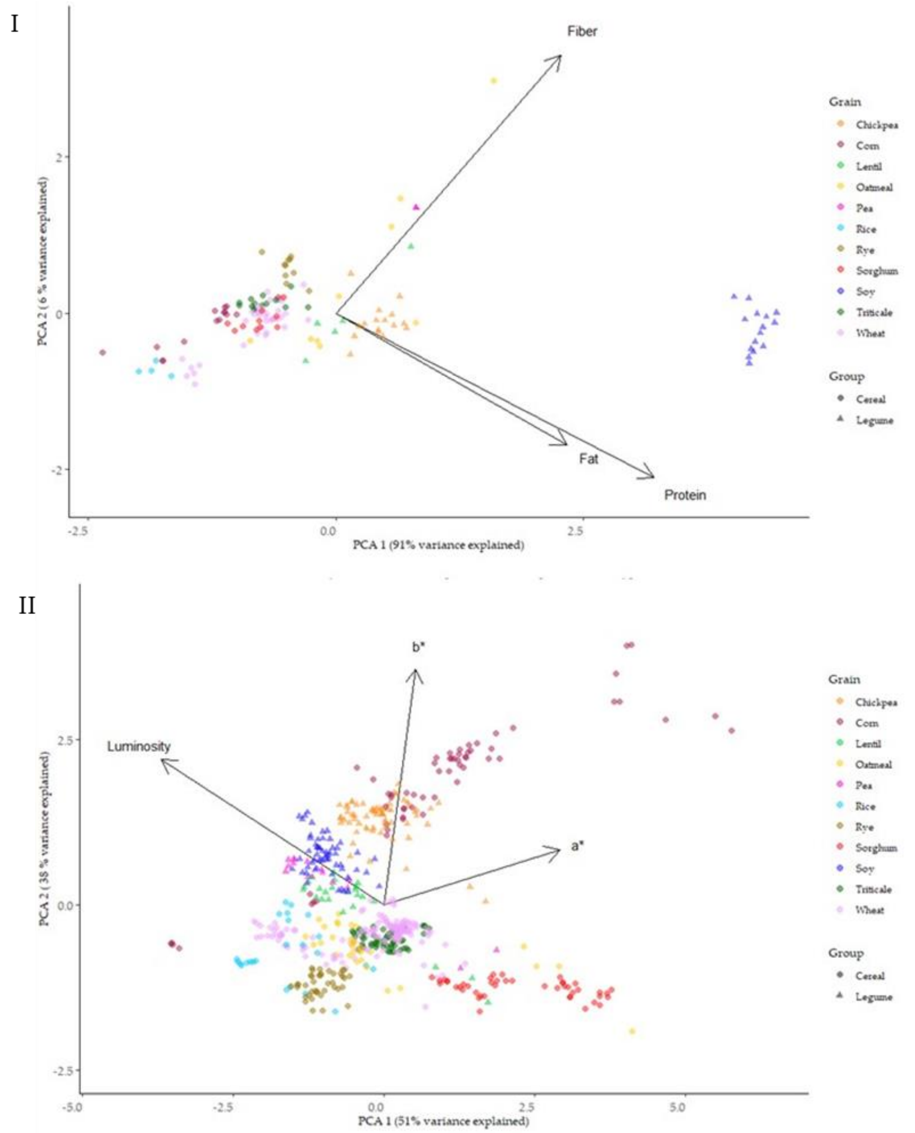

Figure 8. T-distributed Stochastic Neighbor Embedding (T-SNE) distribution of type of grains based on their antioxidant parameters (TP, ORAC and FRAP).

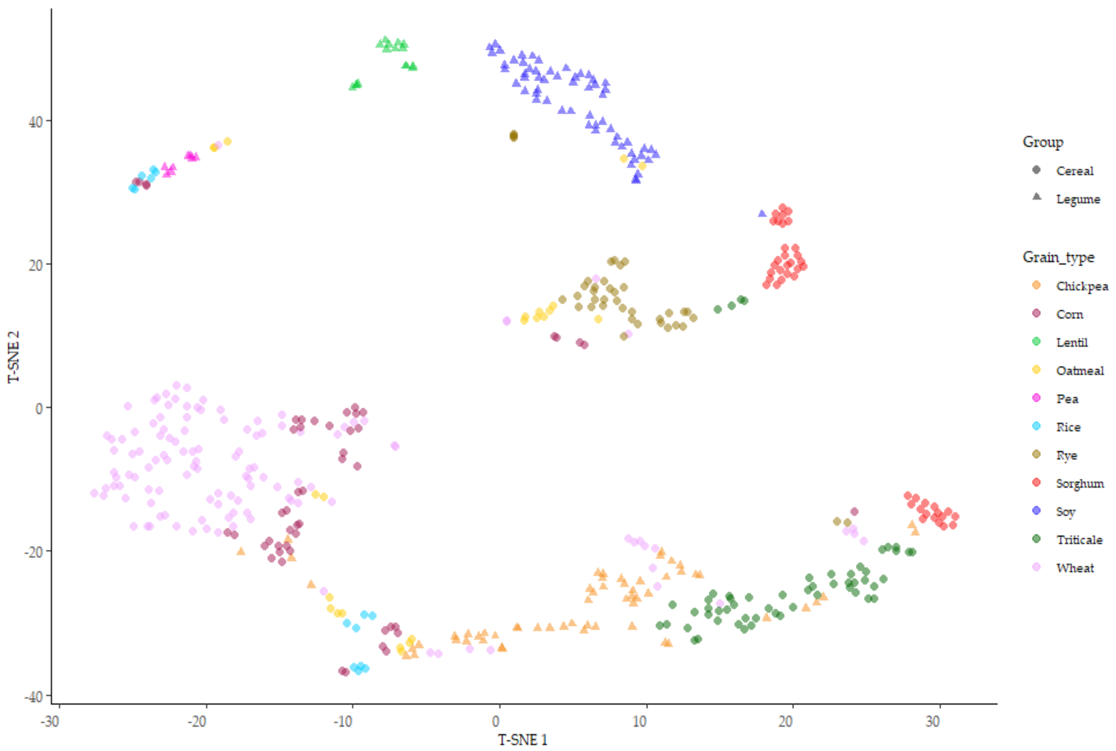

Figure 9. Level curves of Total Phenols, ORAC and FRAP, predicted with the first level model for the series according to baking temperature.

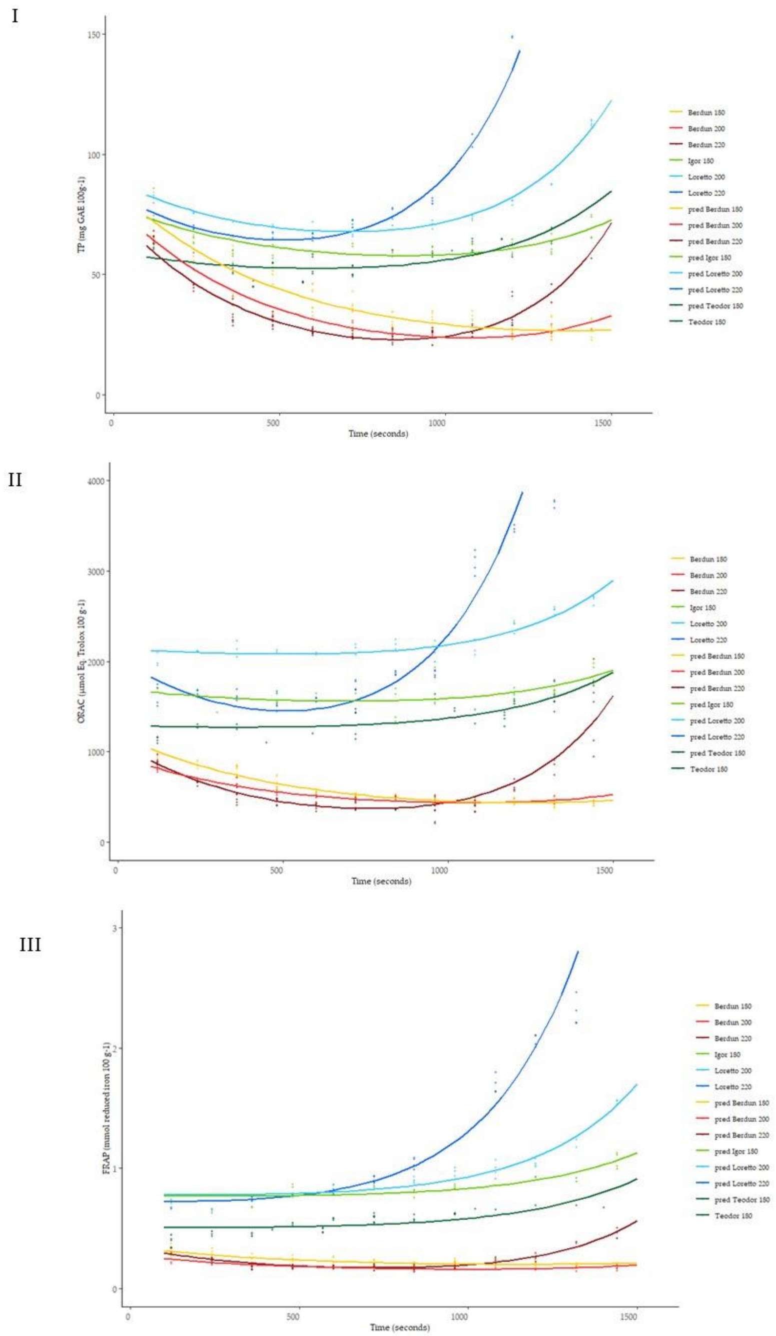

Figure 10. Curves of the level of TP(I-III), ORAC (IV-VI) and FRAP (VII-IX) predicted with the second level models for the series according to baking temperature.

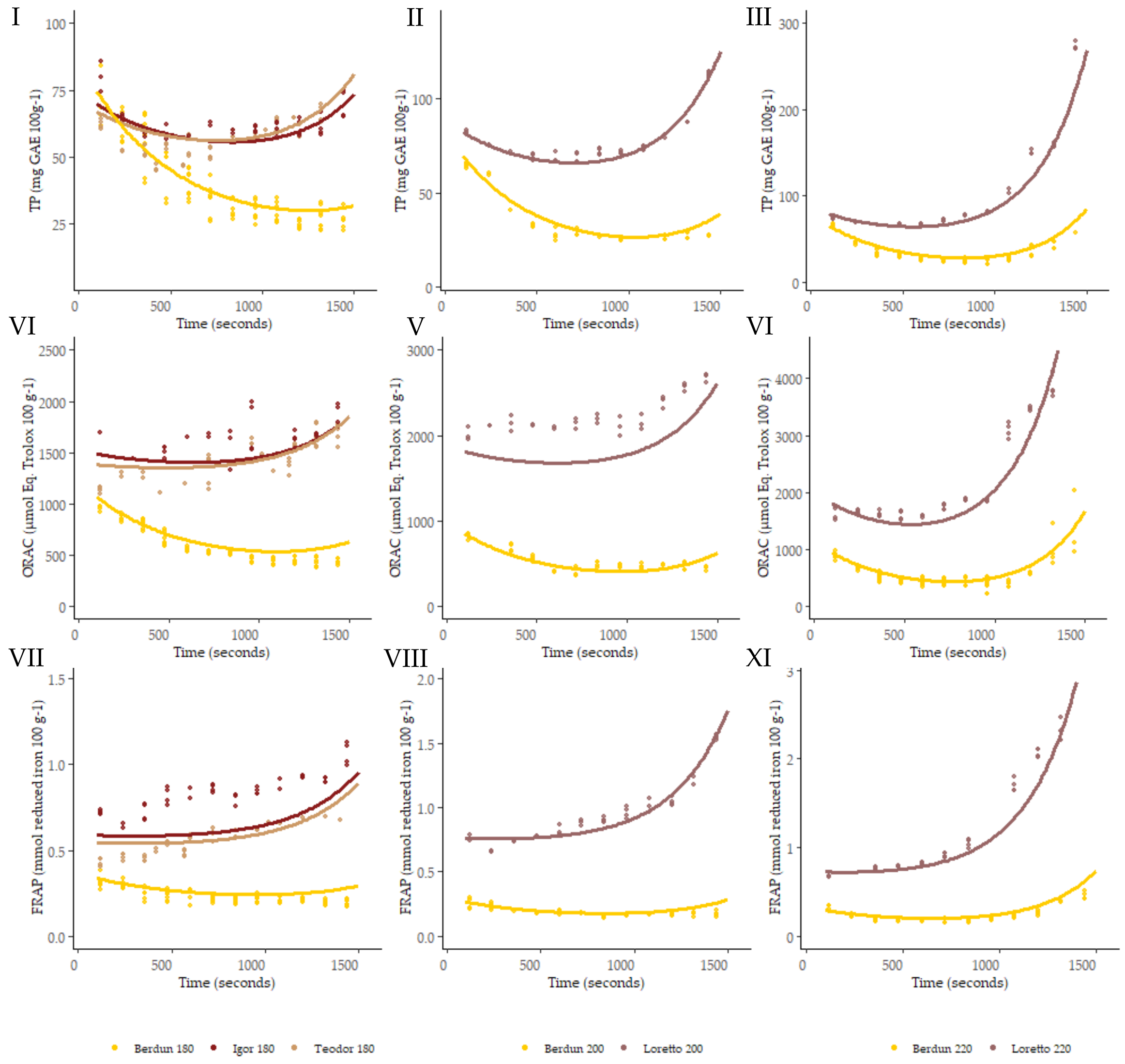

Figure 11. Prediction curves for Total phenol content (TP) based on different baking temperatures on wheat (I) and rye (II).

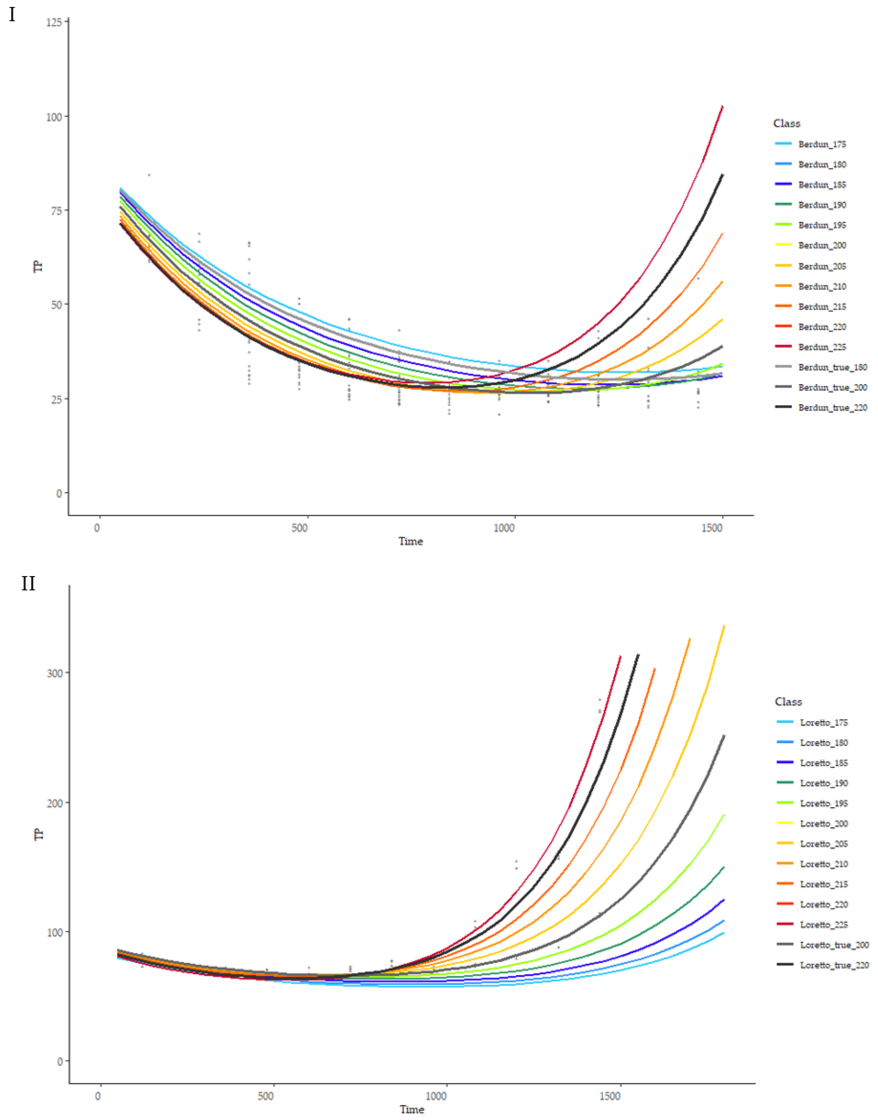

Figure 12. Prediction curves for Total phenol content (TP) based on different protein content (g 100 g^{-1}) on wheat (I) and rye (II).

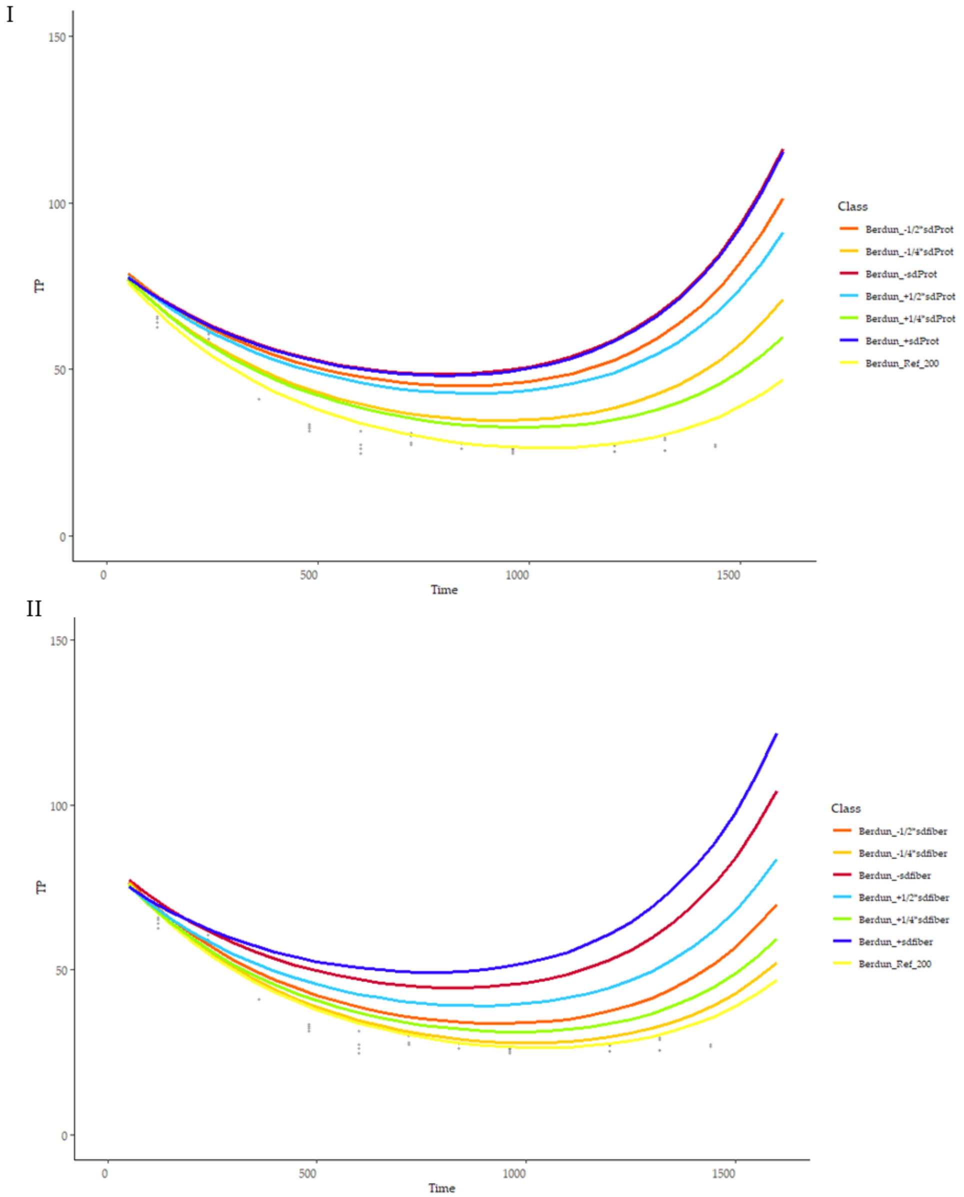

Figure 13. Prediction curves for Total phenol content (TP) based on different fiber (g 100 g^{-1}) content on wheat (I) and rye (II).

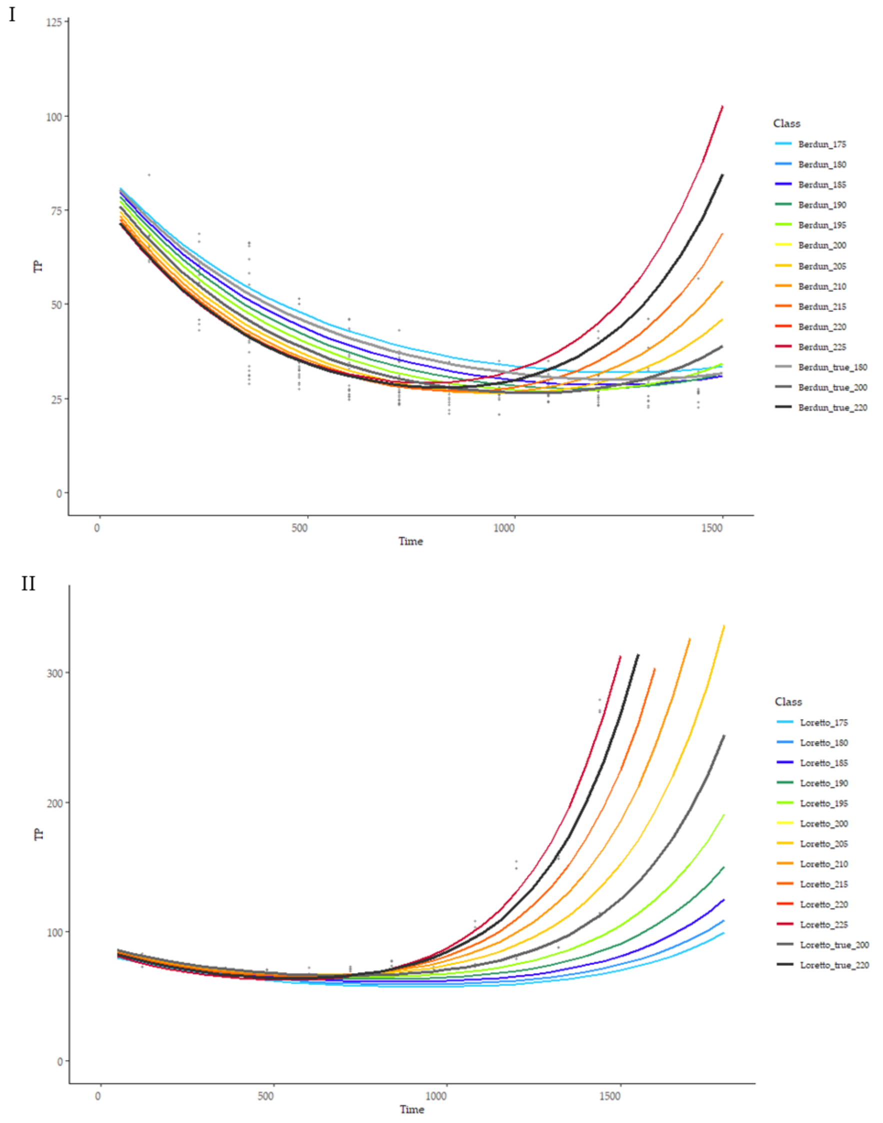

Figure 14. Schematic representation of the global model.

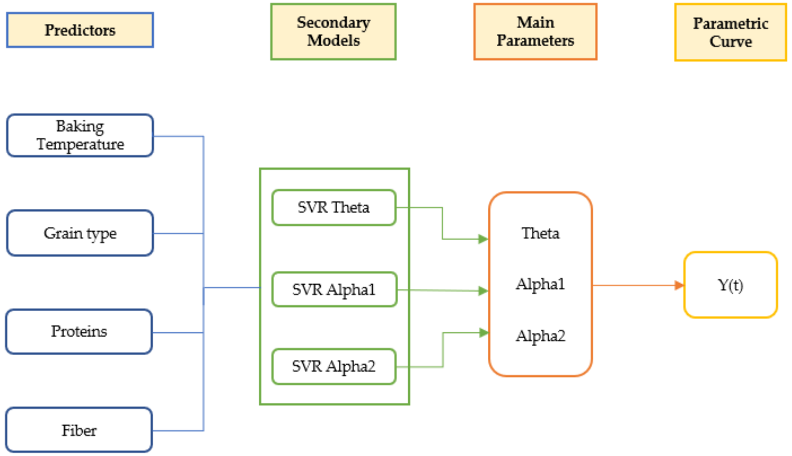

Table 1. Values corresponding to the parameters theta, alpha1 and alpha2 of TP model.

Setting	TP			ORAC			FRAP	
	Theta	Alpha1	Alpha2	Theta	Alpha1	Alpha2	Theta	Alpha1
Berdun 180 °C	0.605	1.985	0.100	0.291	0.826	0.117	0.296	0.297
Igor 180 °C	1.497	0.850	0.573	0.317	0.588	0.106	0.231	0.246
Teodor 180 °C	1.391	0.375	1.013	0.112	0.926	0.566	0.186	0.409
Berdun 200 °C	0.431	1.960	0.265	1.875	0.134	0.564	1.285	0.045
Loretto 200 °C	1.717	0.938	1.022	0.873	1.016	3.034	1.131	0.090
Berdun 220 °C	0.309	1.976	0.566	1.371	0.220	0.450	1.278	0.034
Loretto 220 °C	1.462	1.020	2.516	1.140	0.062	0.686	0.836	0.025

Table 2. R^2 of SRV models.

Temperature	Variety	R^2_TP	R^2_ORAC	R^2_FRAP
180 °C	Berdun	0.807	0.655	-0.372
180 °C	Teodor	0.021	0.500	0.387
180 °C	Igor	0.396	-0.404	-2.081
200 °C	Berdun	0.893	0.716	-0.266
200 °C	Loretto	0.952	-2.051	0.935
220 °C	Berdun	0.632	0.712	0.423
220 °C	Loretto	0.919	0.844	0.881

Supplementary Figure 1. Linear correlation graph of the proximal composition (protein, fiber and fat), colorimetry Luminosity, A and B) and antioxidant parameters (TP, ORAC and FRAP).

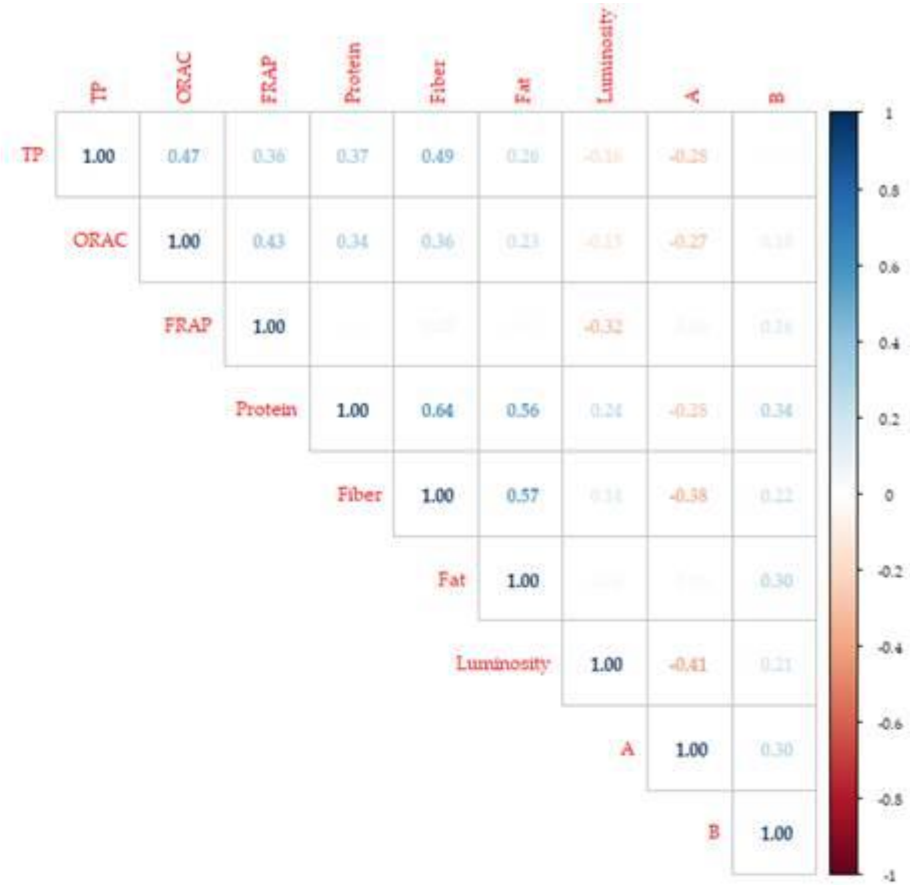

Supplementary Figure 2. Residues of the level of TP (I-III), ORAC (IV-VI) and FRAP (VII-IX) predicted with the second level models for the series according to baking temperature.

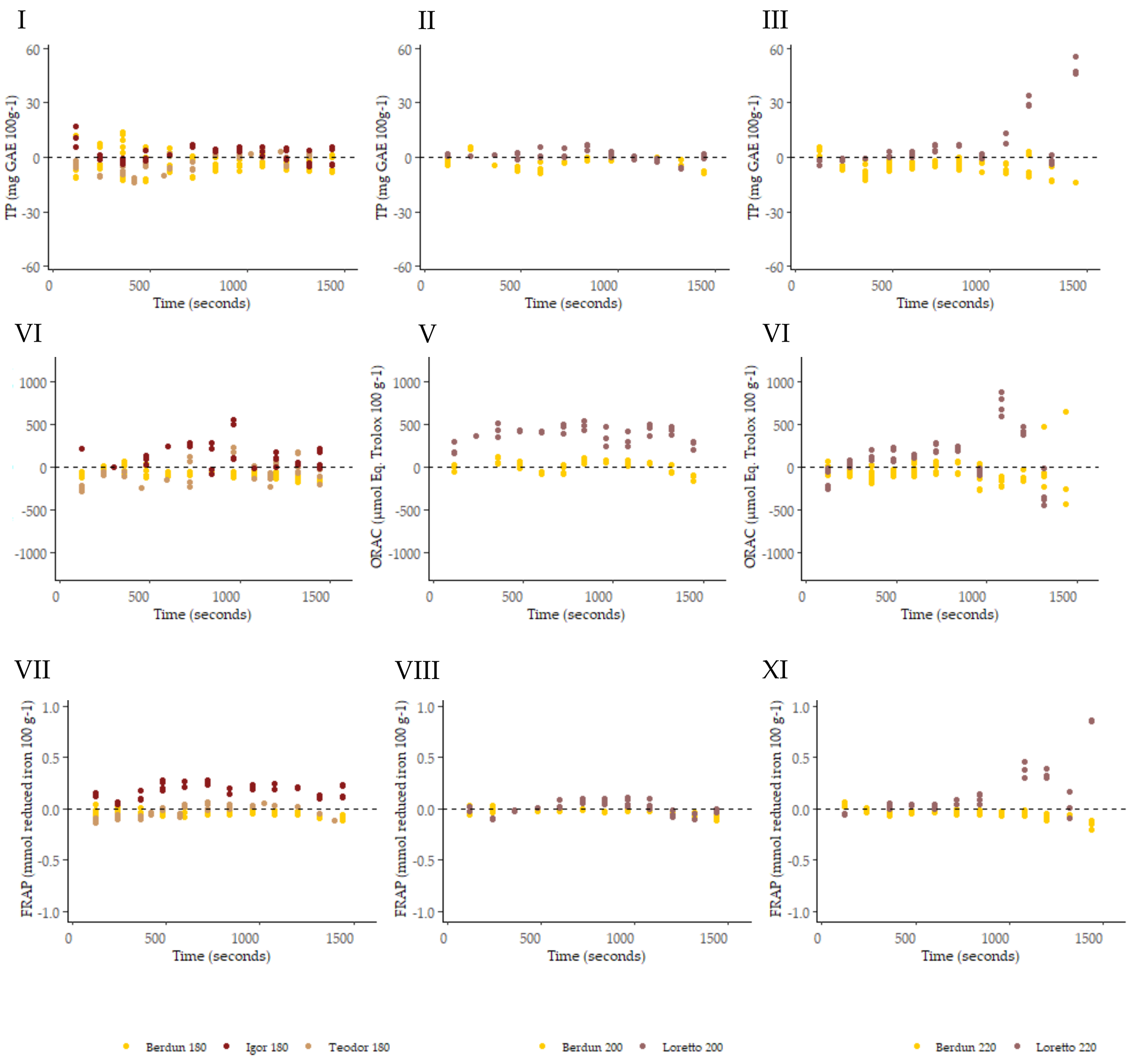

Supplementary Table 1. Silhouette coefficients for each grain type and antioxidant parameter.

Grain type	TP Silhouette	ORAC Silhouette	FRAP Silhouette
Chickpea	-0.106	-0.232	-0.0355
Corn	-0.426	-0.225	-0.731
Lentil	0.633	0.364	-0.717
Oatmeal	-0.585	-0.498	-0.897
Pea	-0.559	-0.377	0.707
Rice	0.170	-0.464	-0.666
Rye	0.276	-0.480	0.132
Sorghum	-0.490	-0.388	0.166
Soy	0.490	-0.218	-0.363
Triticale	0.203	0.334	0.542
Wheat	-0.0333	0.0155	-0.717

Supplementary Table 2. Training dataset for the SVM model that predicts the value of theta in TP modelling.

Variety	Temp_scaled	Grain	Prot_scaled	Fibre_scaled	Theta	IC 95%
Berdun 180 ° C	10.251	0	-0.502	-1.984	0.605	[0.507, 0.685]
Berdun 200 ° C	11.390	0	-0.502	-1.984	0.431	[0.289, 0.575]
Berdun 220 ° C	12.529	0	-0.502	-1.984	0.309	[0.214, 0.406]
Loretto 200 ° C	11.390	1	-1.314	1.272	1.717	[1.583, 1.848]
Loretto 220 ° C	12.529	1	-1.314	1.272	1.462	[1.340, 1.584]
Igor 180 ° C	10.251	1	-0.170	0.778	1.497	[1.329, 1.667]
Teodor 180 ° C	10.251	1	-0.382	1.084	1.391	[1.237, 1.534]

Supplementary Table 3. Training dataset for the SVM model that predicts the value of alpha1 in TP modelling.

Variety	Temp_scaled	Grain	Prot_scaled	Fibre_scaled	Alpha1	IC 95%
Berdun 180 ° C	10.251	0	-0.502	-1.984	1.985	[1.771, 2.214]
Berdun 200 ° C	11.390	0	-0.502	-1.984	1.960	[1.630, 2.290]
Berdun 220 ° C	12.529	0	-0.502	-1.984	1.976	[1.706, 2.241]
Loretto 200 ° C	11.390	1	-1.314	1.272	0.938	[0.599, 1.276]
Loretto 220 ° C	12.529	1	-1.314	1.272	1.020	[0.718, 1.330]
Igor 180 ° C	10.251	1	-0.170	0.778	0.850	[0.500, 1.200]
Teodor 180 ° C	10.251	1	-0.382	1.084	0.375	[0.068, 0.713]

Supplementary Table 4. Training dataset for the SVM model that predicts the value of alpha2 in TP modelling.

Variety	Temp_scaled	Grain	Prot_scaled	Fibre_scaled	Alpha2	IC 95%
Berdun 180 ° C	10.251	0	-0.502	-1.984	0.100	[0.006, 0.269]
Berdun 200 ° C	11.390	0	-0.502	-1.984	0.265	[0.093, 0.442]
Berdun 220 ° C	12.529	0	-0.502	-1.984	0.566	[0.460, 0.670]
Loretto 200 ° C	11.390	1	-1.314	1.272	1.022	[0.869, 1.176]
Loretto 220 ° C	12.529	1	-1.314	1.272	2.516	[2.430, 2.605]
Igor 180 ° C	10.251	1	-0.170	0.778	0.573	[0.293, 0.853]
Teodor 180 ° C	10.251	1	-0.382	1.084	1.013	[0.657, 1.387]

Supplementary Table 5. Cost and R^2 for TP, ORAC and FRAP SVM models.

Model	Cost	R^2
SVR Theta TP	1	0.98
SVR Alpha1 TP	2	0.96
SVR Alpha2 TP	3	0.93
SVR Theta ORAC	2	0.91
SVR Alpha1 ORAC	2.5	0.94
SVR Alpha2 ORAC	3.5	0.90
SVR Theta FRAP	2.5	0.92
SVR Alpha1 FRAP	2	0.94
SVR Alpha2 FRAP	2.5	0.92

Supplementary Table 6. Values corresponding to the main parameters of the ORAC model.

Variety	Theta	IC 95%	Alpha1	IC 95%	Alpha2	IC 95%
Berdun 180 °C	0.290	[0.172, 0.385]	0.826	[0.580, 1.103]	0.117	[0.006, 0.359]
Berdun 200 °C	0.317	[0.175, 0.437]	0.588	[0.267, 0.930]	0.106	[0.006, 0.308]
Berdun 220 °C	0.112	[0.020, 0.215]	0.926	[0.677, 1.156]	0.566	[0.445, 0.678]
Loretto 200 °C	1.875	[1.733, 1.989]	0.134	[0.006, 0.449]	0.564	[0.377, 0.765]
Loretto 220 °C	0.873	[0.738, 1.010]	1.016	[0.685, 1.352]	3.034	[2.909, 3.160]
Igor 180 °C	1.371	[1.181, 1.511]	0.220	[0.010, 0.699]	0.451	[0.127, 0.813]
Teodor 180 °C	1.140	[1.014, 1.250]	0.062	[0.002, 0.268]	0.686	[0.405, 0.978]

Supplementary Table 7. Values corresponding to the main parameters of the FRAP model.

Variety	Theta	IC 95%	Alpha1	IC 95%	Alpha2	IC 95%
Berdun 180 °C	0.296	[0.215, 0.360]	0.297	[0.133, 0.474]	0.059	[0.003, 0.206]
Berdun 200 °C	0.231	[0.125, 0.315]	0.246	[0.040, 0.489]	0.061	[0.003, 0.191]
Berdun 220 °C	0.186	[0.099, 0.272]	0.409	[0.203, 0.615]	0.292	[0.202, 0.385]
Loretto 200 °C	1.285	[1.203, 1.358]	0.045	[0.002, 0.207]	1.020	[0.895, 1.149]
Loretto 220 °C	1.131	[1.041, 1.208]	0.090	[0.004, 0.335]	2.658	[2.581, 2.738]
Igor 180 °C	1.278	[1.199, 1.346]	0.034	[0.001, 0.159]	0.680	[0.478, 0.878]
Teodor 180 °C	0.836	[0.767, 0.899]	0.025	[0.001, 0.127]	0.755	[0.430, 1.086]
